# Management of Vulnerable Patients Hospitalized for COVID-19 With Remdesivir: A Retrospective Comparative Effectiveness Study of Mortality in US Hospitals

**DOI:** 10.1093/cid/ciae512

**Published:** 2024-10-19

**Authors:** Essy Mozaffari, Aastha Chandak, Mark Berry, Paul E Sax, Paul Loubet, Yohei Doi, Alpesh N Amin, Neera Ahuja, Veronika Müller, Roman Casciano, Martin Kolditz

**Affiliations:** Medical Affairs, Gilead Sciences, Foster City, California, USA; Evidence and Access, Certara, New York City, New York, USA; Medical Affairs, Gilead Sciences, Foster City, California, USA; Division of Infectious Diseases, Brigham and Women's Hospital, Boston, Massachusetts, USA; Department of Infectious and Tropical Diseases, Centre Hospitalier Universitaire de Nimes, Nimes, France; Departments of Microbiology and Infectious Diseases, Fujita Health University School of Medicine, Toyoake, Japan; Division of Infectious Diseases, University of Pittsburgh School of Medicine, Pittsburgh, Pennsylvania, USA; Department of Medicine, School of Medicine, University of California Irvine, Irvine, California, USA; Department of Internal Medicine, Stanford University School of Medicine, Stanford, California, USA; Department of Pulmonology, Semmelweis University, Budapest, Hungary; Evidence and Access, Certara, New York City, New York, USA; Medical Department I, University Hospital Carl Gustav Carus of TU Dresden, Dresden, Saxony, Germany

**Keywords:** real-world data, Omicron, COVID-19, SARS-CoV-2, remdesivir, elderly, pneumonia, hospitalization, data science, propensity score, real-world data, comorbidity, real-world evidence

## Abstract

**Background:**

Coronavirus disease 2019 (COVID-19) remains a major public health concern, with continued resurgences of cases and substantial risk of mortality for hospitalized patients. Remdesivir has become standard-of-care for hospitalized COVID-19 patients. Given the continued evolution of the disease, clinical management of COVID-19 relies on evidence from the current endemic period.

**Methods:**

Using the PINC AI Healthcare Database, remdesivir effectiveness was evaluated among adults hospitalized with primary diagnosis of COVID-19 between December 2021 and February 2024. Three cohorts were analyzed: adults (≥18 years), elderly (≥65 years), and those with documented COVID-19 pneumonia. Analyses were stratified by oxygen requirements. Patients who received remdesivir were matched to those who did not receive remdesivir using propensity score matching. Cox proportional hazards models were used to examine in-hospital mortality.

**Results:**

169 965 adults hospitalized for COVID-19 were included, of whom 94 129 (55.4%) initiated remdesivir in the first 2 days of hospitalization. Remdesivir was associated with significantly lower mortality rate compared to no remdesivir among patients with no supplemental oxygen charges (adjusted HR [95% CI]: 14-day, 0.75 [.69–.82]; 28-day, 0.77 [.72–.83]) and those requiring supplemental oxygen: 14-day, 0.76 [.72–.81]; 28-day, 0.79 [.74–.83]; *P* < .0001 for all). Similar findings were observed for elderly patients and those hospitalized with COVID-19 pneumonia.

**Conclusions:**

This evidence builds on what has been learned from randomized controlled trials from the pandemic era to inform clinical practices. Remdesivir was associated with significant reduction in mortality for hospitalized patients including the elderly and those with COVID-19 pneumonia.

## Teaser

Complementing the evidence from randomized clinical trials, real-world evidence from the Omicron era demonstrates that initiation of remdesivir in patients hospitalized for coronavirus disease 2019 saved lives compared with no initiation of remdesivir, reconfirming clinical practice guideline recommendations.

Coronavirus disease 2019 (COVID-19), an infectious disease caused by the severe acute respiratory syndrome coronavirus 2 virus (SARS-CoV-2), was officially recognized by the World Health Organization (WHO) as a pandemic in March 2020 [1, 2] and was reclassified as an endemic in May 2023 [3]. COVID-19 remains a major public health concern, with large resurgences, even in geographic locales where vaccination has been widespread. In 2024, the WHO considered COVID-19 a continuing threat to the public and to healthcare systems [4]. Prolonged hospitalization, as well as a substantial risk of mortality due to severe COVID-19, remain the norm today for certain patient segments, for example, the elderly, those with significant comorbidities, and patients with COVID-19 pneumonia [5, 6].

Fortunately, clinical management of COVID-19 has evolved since the start of the pandemic due to availability of antiviral treatments. In the pivotal double-blind, randomized, placebo-controlled Adaptive COVID-19 Treatment Trial (ACTT-1), remdesivir was found to be superior to placebo in shortening the time to recovery and reducing all-cause mortality in patients hospitalized for COVID-19 [7]. That study led to emergency use authorization on 1 May 2020 for remdesivir in adults and children hospitalized for COVID-19. Subsequently, remdesivir became the first United States (US) Food and Drug Administration–approved antiviral for COVID-19 on 22 October 2020 [8]. Since then, numerous additional studies, including real-world studies, have reinforced the initial findings of ACTT-1 and extended the evidence in support of remdesivir’s effectiveness [9–12], which neither the ACTT-1 study nor other randomized clinical trials (RCTs) of remdesivir were sufficiently powered to address [13, 14].

Treatment guidelines were initially crafted based on the RCTs conducted early in the pandemic, but only the National Institutes of Health guidelines have undergone recent updates [15] to include the most current body of evidence, including real-world evidence [16]. These COVID-19 treatment guidelines have recently been retired. While RCTs for hospitalized COVID-19 patients have been foundational in assessing treatment efficacy early in the course of the pandemic, they were not powered sufficiently to address certain patient subgroups [13, 14] and may no longer be feasible to perform in our most vulnerable patient groups for both practical and ethical reasons. Evidence derived from real-world data therefore represents an opportunity to complement the RCTs with contemporaneous evidence on treatment effectiveness realized in everyday clinical practice for the full spectrum of patients hospitalized for COVID-19.

Our research objective was to provide comprehensive and up-to-date evidence for management of patients hospitalized for COVID-19 with remdesivir. This real-world comparative effectiveness study examined the effectiveness of remdesivir with respect to inpatient mortality during the Omicron predominant period across the full spectrum of patients hospitalized for COVID-19. This study adds to previous observational studies by providing contemporaneous information regarding the effectiveness of remdesivir, specifically with the evolving Omicron subvariants that were not examined in prior study periods. Furthermore, another major contribution of this study is evidence among the vulnerable patient populations who are elderly and those who are admitted with COVID-19 pneumonia.

## METHODS

### Study Design and Data Source

In this retrospective, comparative effectiveness study, we used patient-level data from the PINC AI Healthcare Database (PHD, formerly Premier Healthcare Database; www.pinc-ai.com), a large, geographically diverse, Health Insurance Portability and Accountability Act–compliant, all-payer hospital administrative billing database that covers approximately 25% of hospitalizations from 48 states in the US. The database includes patient-level demographic data, disease state, diagnoses at admission and discharge, as well as hospital characteristics [17]; <1% of patient records have missing information across all data elements recorded, and <0.01% have missing information for key elements such as demographics and diagnostic information.

The study period was between December 2021 and February 2024 (Omicron-predominant based on the circulating SARS-CoV-2 variants during this period in the US) and stratified by early Omicron period (December 2021 to December 2022) and late Omicron period (January 2023 to February 2024).

### Study Population

The study included patients aged ≥18 years hospitalized with a primary discharge diagnosis of COVID-19 (*International Classification of Diseases, 10th revision, Clinical Modification* [ICD-10-CM] code U07.1) that was also flagged as “present on admission.” The use of the COVID-19 diagnosis code (U07.1) has been previously validated in the PHD [18]. Only the first COVID-19 hospitalization for a patient was included. Patients were categorized according to their oxygen supplementation requirements within the first 2 days of their hospitalization. Patients who did not require supplemental oxygen during the first 2 days of hospitalization (a proxy for disease severity) were identified by an absence of any oxygen supply–related charges (no supplemental oxygen charges [NSOc]) and absence of any charges for devices for low-flow oxygen, high-flow oxygen, noninvasive ventilation, invasive mechanical ventilation, and extracorporeal membrane oxygenation (ECMO). Because some hospitals do not bill separately for supplemental oxygen supply or devices and instead include these charges in room charges, it may not be possible to identify supplemental oxygen use in such hospitals. Accordingly, only patients admitted to hospitals that reported separate charges for supplemental oxygen were included in the study. Prior research has shown that additional restrictive criteria such as including only those patients admitted to hospitals that reported separate charges for supplemental oxygen every 6 months or every quarter of the study period did not impact the study findings [19].

Exclusion criteria included pregnancy, incomplete data fields, transfer from another hospital or hospice, transfer to another hospital, admission for elective procedures, discharge or death during the baseline period (first 2 days of hospitalization), patients who required ECMO at baseline, and initiation of remdesivir after the first 2 days of hospitalization.

Three patient cohorts were analyzed: patients ≥18 years of age, elderly patients (aged ≥65 years, 65–75 years, 75–84 years, and ≥85 years), and those with COVID-19 pneumonia (ICD-10-CM code J12.82).

### Study Outcome and Covariates

The primary end point of the study was all-cause inpatient mortality (defined as a discharge status of “expired” or “hospice”) at 14 and 28 days after the first 2 days of hospitalization during which treatment with remdesivir was ascertained. Patients were followed from day 3 of hospitalization (ie, after the baseline period during which remdesivir treatment and baseline supplemental oxygen requirements were ascertained) until death or the end of follow-up. Patients discharged alive and not to a hospice setting were censored at 14 and 28 days to assess 14- and 28-day mortality, respectively.

All baseline variables were examined within the first 2 days of hospitalization. This definition of baseline was chosen since actual time stamps are unavailable in the database; for example, for a patient admitted to the hospital at 23:59, the patient’s Day 2 would start at 00:00. The definition for baseline therefore provided all patients a window of a minimum of 24 hours during which clinical decisions were made and implemented. Baseline covariates included in propensity score (PS) models were demographics (age, gender, race, ethnicity, and primary payor), key comorbidities (obesity, chronic obstructive pulmonary disease [COPD], cardiovascular disease, diabetes, renal disease, and immunocompromising conditions), hospital characteristics (bed size, location [urban, rural, US region], hospital ward on admission (general ward or intensive care unit [ICU]), admission from skilled nursing facility, admission month, other indicators of severity based on admission diagnoses (sepsis and pneumonia), and concomitant COVID-19 treatment at baseline (anticoagulants, corticosteroids, convalescent plasma, baricitinib, or tocilizumab and other oral antivirals; Supplementary Table 1).

Treatment groups included those who received ≥1 dose of remdesivir within 2 days of admission and those who did not receive remdesivir during the duration of hospitalization for COVID-19.

### Statistical Analyses

Demographic and baseline clinical characteristics and other COVID-19 medications were summarized descriptively. The PSs represent the probability of receiving the treatment of interest. In this study, PSs were estimated using separate logistic regression models with exposure to remdesivir as the dependent variable for the 2 supplemental oxygen requirement categories (NSOc vs supplemental oxygen charges [SOc]) and included baseline covariates such as demographics, key comorbid conditions, hospital characteristics, admission diagnoses, hospital ward on admission, and concomitant medications. All covariates were retained in the model irrespective of their *P* value. The 2 separate models were used to ensure valid comparability within the supplemental oxygen groups. Further, separate PSs were computed for each of the 3 patient cohorts analyzed in this study.

Using the derived PSs, distribution of underlying confounders in the 2 treatment groups (remdesivir vs no remdesivir) was balanced using PS matching (PSM) as the primary analysis. To account for differences in hospital COVID-19 management practices, a 1:1 preferential within-hospital matching approach without replacement with a caliper distance of 0.2 times standard deviation of the logit of the PS was implemented. Patients who received remdesivir were matched to patients who did not receive remdesivir in the same hospital within the specified caliper distance in the same age group (18–49, 50–64, and ≥65 years) and admission month group (2- to 3-month blocks of admission month); unmatched patients were then matched to patients who did not receive remdesivir in another remdesivir-using hospital of similar bed size (<200, 200–499, ≥500 beds) within the specified caliper distance in the same age group and admission month group.

The proportional hazard assumption was met for each analysis as assessed through the Kaplan–Meier curve (where the curves did not cross over) and log of negative log plot (which showed parallel lines that did not cross over). A Cox proportional hazards model was used to assess time to 14- and 28-day in-hospital all-cause mortality separately for the 2 time points, and adjusted hazard ratios (aHRs) and 95% confidence intervals (CIs) were derived. The models were adjusted for hospital-level cluster effects using a robust sandwich variance estimator and key covariates of age (as a continuous variable), admission month, and hospital ward on admission (documented bed charges for ICU/step-down unit vs general ward). Treatments with corticosteroids, baricitinib, and tocilizumab after the first 2 days of hospitalization were adjusted for as time-varying covariates. These additional adjustment variables were prespecified to account for any remaining residual confounding among these variables as they were identified to be key covariates that impacted the study outcomes.

### Sensitivity Analyses

Two sensitivity analyses were performed: (1) inverse probability of treatment weighting (IPTW) to PSM [20]; in this approach, extreme PSs <0.05 and >0.95 were trimmed; and (2) examining patients who initiated remdesivir within 2 days of admission vs those who did not initiate remdesivir within 2 days of admission (ie, patients who were never treated with remdesivir or those who were treated after the first 2 days of hospitalization).

## RESULTS

**Figure 1.**
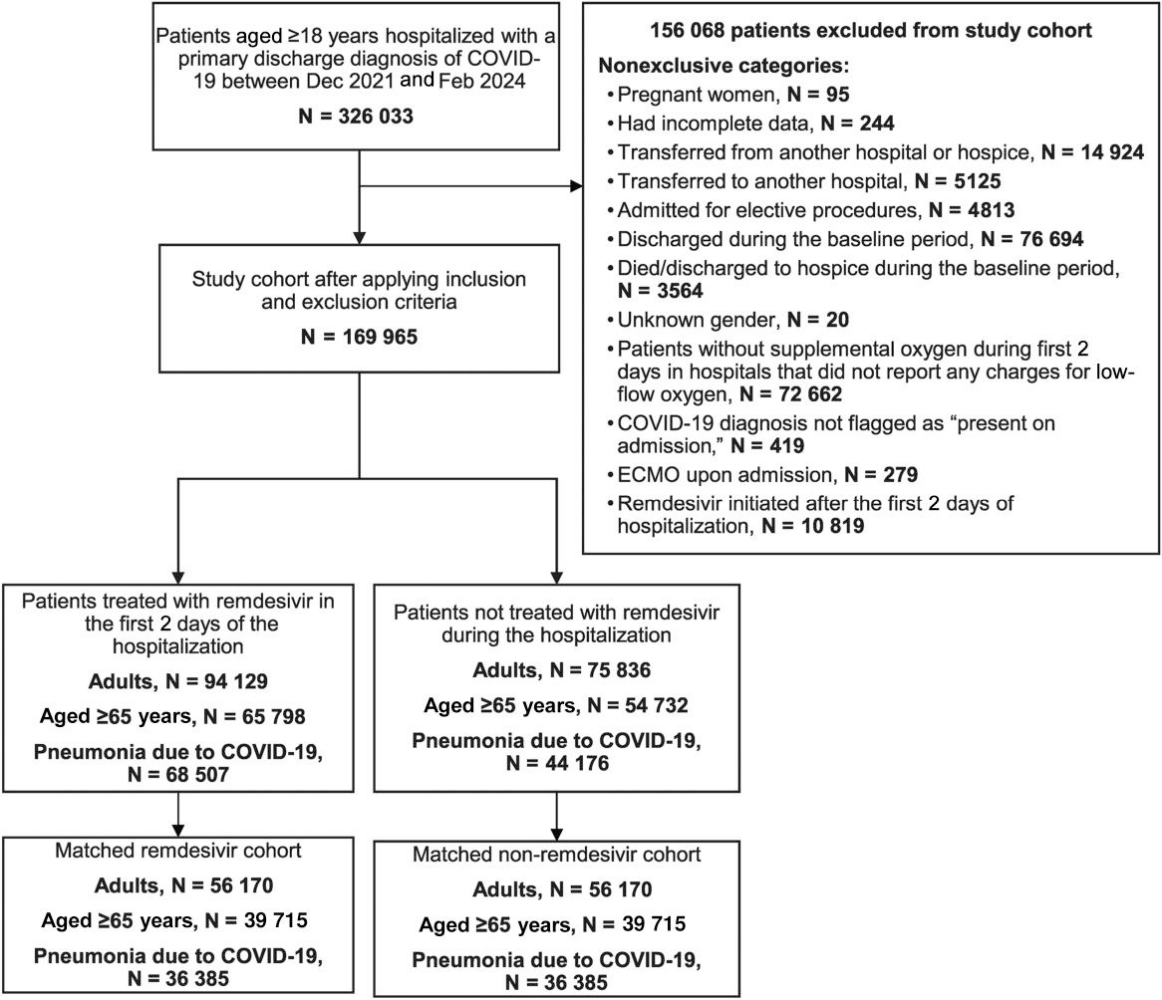
Study flow diagram. Abbreviations: COVID-19, coronavirus disease 2019; ECMO, extracorporeal membrane oxygenation.

In total, 326 033 adult patients hospitalized with a primary discharge diagnosis of COVID-19 flagged as “present-on-admission” were identified during the study period (Figure 1).

### Adult Population

After applying inclusion/exclusion criteria, the population included 169 965 patients of whom 94 129 (55.4%) initiated remdesivir in the first 2 days of hospitalization and 75 836 (44.6%) who were not treated with remdesivir during the hospitalization (Figure 1). Before PSM, most patients in the remdesivir and no-remdesivir cohorts, respectively, were aged ≥65 years (70%, 72%) and had cardiovascular disease (87%, 89%). A lower proportion of patients who received remdesivir compared with those who did not receive remdesivir had renal disease (25%, 34%), while a higher proportion of patients had COPD (38%, 33%) and required supplemental oxygen (56%, 45%). Median [interquartile range, IQR, Q1, Q3] duration of remdesivir use was 5.0 days [3.0, 5.0]. After 1:1 PSM, 56 170 patients who received remdesivir were matched to 56 170 patients who did not receive remdesivir during hospitalization. Characteristics were well balanced after PSM, with all covariates demonstrating an absolute standardized difference of <0.15 (Table 1, Supplementary Figure 1). Baseline demographics and hospital characteristics of adults hospitalized for COVID-19 before and after IPTW are shown in Supplementary Table 2.

**Table 1.** Demographics of Adults Hospitalized for COVID-19 During December 2021–February 2024 Before and After PSM

Characteristic	Before PSM			After PSM		
	No Remdesivir n = 75 836	Remdesivir n = 94 129	SMD	No Remdesivir n = 56 170	Remdesivir n = 56 170	SMD
**Age group, y**						
18–49	6769 (8.9%)	8689 (9.2%)	0.05	4325 (7.7%)	4325 (7.7%)	0.00
50–64	14 335 (18.9%)	19 642 (20.9%)		10 875 (19.4%)	10 875 (19.4%)	
≥65	54 732 (72.2%)	65 798 (69.9%)		40 970 (72.9%)	40 970 (72.9%)	
**Gender**						
Female	38 980 (51.4%)	47 961 (51.0%)	0.01	28 735 (51.2%)	28 718 (51.1%)	0.00
**Race**						
White	56 934 (75.1%)	72 221 (76.7%)	0.09	43 093 (76.7%)	43 231 (77.0%)	0.00
Black	12 148 (16.0%)	12 230 (13.0%)		7986 (14.2%)	7870 (14.0%)	
Asian	1383 (1.8%)	2266 (2.4%)		1096 (2.0%)	1119 (2.0%)	
Other	5371 (7.1%)	7412 (7.9%)		3995 (7.1%)	3950 (7.0%)	
**Ethnicity**						
Hispanic	5931 (7.8%)	10 103 (10.7%)	0.11	4603 (8.2%)	4412 (7.9%)	0.04
Non-Hispanic	64 361 (84.9%)	78 574 (83.5%)		47 988 (85.4%)	48 158 (85.7%)	
Unknown	5544 (7.3%)	5452 (5.8%)		3579 (6.4%)	3600 (6.4%)	
**Primary payor**						
Commercial	9662 (12.7%)	14 670 (15.6%)	0.13	7677 (13.7%)	7425 (13.2%)	0.05
Medicare	55 698 (73.4%)	66 690 (70.8%)		41 287 (73.5%)	41 424 (73.7%)	
Medicaid	6388 (8.4%)	8177 (8.7%)		4385 (7.8%)	4416 (7.9%)	
Other	4088 (5.4%)	4592 (4.9%)		2821 (5.0%)	2905 (5.2%)	
**Admission source**						
Transferred from skilled nursing facility or intermediate care facility	2651 (3.5%)	3589 (3.8%)	0.02	2084 (3.7%)	2006 (3.6%)	0.01
**Hospital size, number of beds**						
<100	6136 (8.1%)	7301 (7.8%)	0.10	4595 (8.2%)	4560 (8.1%)	0.04
100–199	12 194 (16.1%)	16 260 (17.3%)		9193 (16.4%)	9228 (16.4%)	
200–299	16 267 (21.5%)	19 030 (20.2%)		12 100 (21.5%)	11 756 (20.9%)	
300–399	14 330 (18.9%)	15 263 (16.2%)		10 082 (17.9%)	10 006 (17.8%)	
400–499	8532 (11.3%)	10 267 (10.9%)		6298 (11.2%)	6718 (12.0%)	
500+	18 377 (24.2%)	26 008 (27.6%)		13 902 (24.7%)	13 902 (24.7%)	
**Hospital location**						
Urban	66 070 (87.1%)	83 163 (88.4%)	0.04	49 060 (87.3%)	49 071 (87.4%)	0.00
Rural	9766 (12.9%)	10 966 (11.6%)		7110 (12.7%)	7099 (12.6%)	
**Teaching hospital**	31 286 (41.3%)	40 685 (43.2%)	0.04	23 109 (41.1%)	23 257 (41.4%)	0.01
**Region**						
Midwest	19 227 (25.4%)	22 369 (23.8%)	0.15	14 414 (25.7%)	14 077 (25.1%)	0.03
Northeast	8727 (11.5%)	15 532 (16.5%)		7009 (12.5%)	7243 (12.9%)	
South	39 694 (52.3%)	44 582 (47.4%)		28 218 (50.2%)	28 229 (50.3%)	
West	8188 (10.8%)	11 646 (12.4%)		6529 (11.6%)	6621 (11.8%)	
**Comorbid conditions**						
Obesity	20 398 (26.9%)	28 023 (29.8%)	0.06	15 936 (28.4%)	15 831 (28.2%)	0.00
Chronic obstructive pulmonary disease	25 221 (33.3%)	36 133 (38.4%)	0.11	20 341 (36.2%)	20 482 (36.5%)	0.01
Cardiovascular disease	67 117 (88.5%)	81 565 (86.7%)	−0.06	49 590 (88.3%)	49 601 (88.3%)	0.00
Diabetes	30 277 (39.9%)	36 340 (38.6%)	−0.03	22 181 (39.5%)	22 173 (39.5%)	0.00
Renal disease	25 492 (33.6%)	23 827 (25.3%)	−0.18	17 629 (31.4%)	17 351 (30.9%)	0.01
Immunocompromising condition	12 236 (16.1%)	16 730 (17.8%)	0.04	9578 (17.1%)	9573 (17.0%)	0.00
Cancer	5125 (6.8%)	7163 (7.6%)	0.03	3994 (7.1%)	4055 (7.2%)	0.00
**Hospital ward on admission**						
General ward	63 560 (83.8%)	75 932 (80.7%)	0.08	46 594 (83.0%)	46 759 (83.2%)	0.01
Intensive care unit/step-down unit	12 276 (16.2%)	18 197 (19.3%)		9576 (17.0%)	9411 (16.8%)	
**Key diagnosis on admission**						
Sepsis	365 (0.5%)	342 (0.4%)	−0.02	246 (0.4%)	251 (0.4%)	0.00
Pneumonia	4622 (6.1%)	6036 (6.4%)	0.01	3676 (6.5%)	3664 (6.5%)	0.00
**Other COVID-19 treatments at baseline**						
Anticoagulants	53 852 (71.0%)	73 580 (78.2%)	0.16	41 901 (74.6%)	42 027 (74.8%)	0.01
Convalescent plasma	30 (0.0%)	105 (0.1%)	0.54	28 (0.0%)	24 (0.0%)	0.00
Corticosteroids	47 557 (62.7%)	80 504 (85.5%)	0.03	43 694 (77.8%)	43 677 (77.8%)	0.00
Baricitinib	4103 (5.4%)	5005 (5.3%)	0.08	3396 (6.0%)	3340 (5.9%)	0.00
Tocilizumab	1863 (2.5%)	3543 (3.8%)	0.00	1700 (3.0%)	1705 (3.0%)	0.00
Oral antivirals	1306 (1.7%)	204 (0.2%)	−0.15	191 (0.3%)	156 (0.3%)	0.01
**Baseline supplemental oxygen requirements**						
No supplemental oxygen charges	41 894 (55.2%)	41 013 (43.6%)	0.27	27 704 (49.3%)	27 704 (49.3%)	0.00
Low-flow oxygen	20 794 (27.4%)	31 808 (33.8%)		17 682 (31.5%)	17 682 (31.5%)	
High-flow oxygen/noninvasive ventilation	10 598 (14.0%)	18 887 (20.1%)		9277 (16.5%)	9277 (16.5%)	
Invasive mechanical ventilation	2550 (3.4%)	2421 (2.6%)		1507 (2.7%)	1507 (2.7%)	
**Omicron period**						
Early (Dec 2021–Dec 2022)	54 372 (71.7%)	66 484 (70.6%)	0.02	40 439 (72.0%)	40 439 (72.0%)	0.00
Late (Jan 2023–Feb 2024)	21 464 (28.3%)	27 645 (29.4%)		15 731 (28.0%)	15 731 (28.0%)	

Abbreviations: COVID-19, coronavirus disease 2019; PSM, propensity score matching; SMD, standardized mean difference.

**Figure 2.**
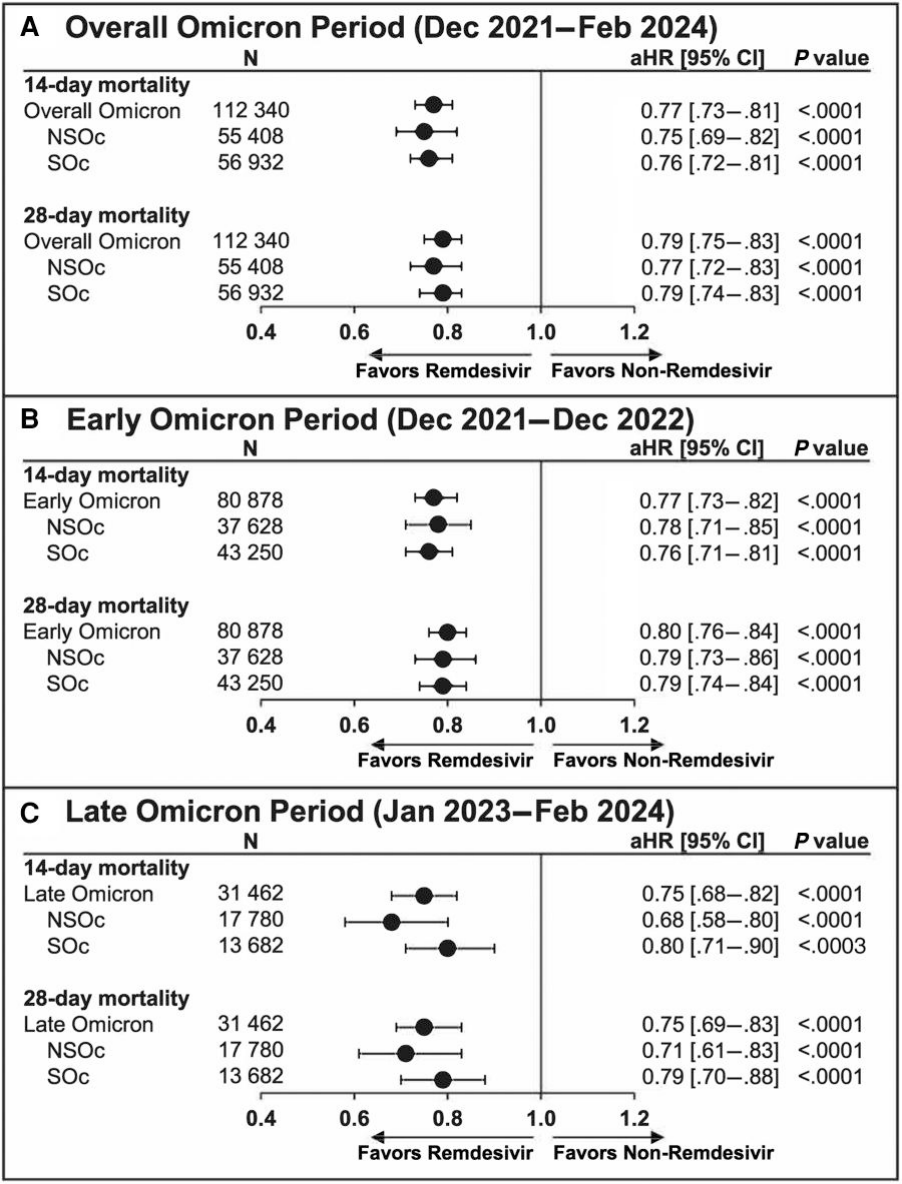
The 14- and 28-day mortality in adults hospitalized for coronavirus disease 2019 (COVID-19) who received and did not receive remdesivir: propensity score matching. Cox proportional hazards model used to derive estimates adjusted for age, admission month, hospital ward upon admission (intensive care unit vs general ward), and time-varying treatment with other COVID-19 medications (baricitinib, tocilizumab, oral antivirals). (*A*), Overall Omicron period. (*B*), Early Omicron period. (*C*), Late Omicron period. Abbreviations: aHR, adjusted hazard ratio; CI, confidence interval; NSOc, no supplemental oxygen charges; SOc, supplemental oxygen charges.

Unadjusted mortality risk was 7.2% vs 9.0% at 14 days and 9.5% vs 11.5% at 28 days for the remdesivir vs no-remdesivir groups, respectively. After adjusting for baseline and clinical covariates, remdesivir was associated with a significantly lower 14- and 28-day mortality rate compared with no remdesivir (aHR [95% CI]: 0.77 [.73–.81] and 0.79 [.75–.83], respectively; *P* < .0001; Figure 2*A*). These findings were consistently observed for the early (Figure 2*B*) and late Omicron periods (Figure 2*C*).

For the NSOc subpopulation, unadjusted mortality risk for the entire Omicron period was 4.5% vs 5.6% at 14 days and 5.7% vs 6.8% at 28 days for the remdesivir vs no-remdesivir groups, respectively. After adjusting for baseline and clinical covariates, remdesivir was associated with a significantly lower 14- and 28-day mortality rate compared with no remdesivir (aHR [95% CI]: 0.75 [.69–.82] and 0.77 [.72–.83], respectively; *P* < .0001; Figure 2*A*). Similar findings for early and late Omicron periods are shown in Figures 2*B* and 2*C*, respectively.

For the SOc subpopulation, unadjusted mortality risk for the entire Omicron period was 9.8% vs 12.3% at 14 days and 13.2% vs 16.0% at 28 days for the remdesivir vs no-remdesivir groups, respectively. After adjusting for baseline and clinical covariates, remdesivir was associated with a significantly lower 14- and 28-day mortality rate compared with no remdesivir (aHR [95% CI]: 0.76 [.72–.81] and 0.79 [.74–.83], respectively; *P* < .0001; Figure 2*A*). Similar to the findings listed above, early and late Omicron periods are shown in Figures 2*B* and 2*C*, respectively.

These findings were also consistent for the sensitivity analyses that used IPTW (Supplementary Table 3) and for the sensitivity analysis that compared remdesivir initiation in the first 2 days of admission vs no remdesivir initiation in the first 2 days of admission (Supplementary Table 4).

### Elderly Population

The elderly population included 120 530 patients of whom 65 798 (54.6%) initiated remdesivir in the first 2 days of hospitalization and 54 732 (45.4%) who were not treated with remdesivir during the hospitalization (Figure 1). Before PSM, there was a relatively even distribution of patients in the remdesivir and no-remdesivir cohorts, respectively, who were aged ≥65–74 years (34%, 32%), 75–84 years (38%, 38%), and ≥85 years (28%, 30%) and with cardiovascular disease (93%, 94%). A lower proportion of patients who received remdesivir compared with those who did not receive remdesivir had renal disease (30%, 38%), while a higher proportion had COPD (40%, 34%) and required supplemental oxygen (54%, 43%). Median duration of remdesivir use was 5.0 days [IQR Q1, Q3 3.0, 5.0]. After 1:1 PSM, 39 715 patients treated with remdesivir were matched to 39 715 patients not treated with remdesivir during hospitalization. Characteristics were well balanced after PSM, with all covariates demonstrating an absolute standardized difference of <0.15 (Supplementary Table 5, Supplementary Figure 2). Baseline demographics and hospital characteristics of the elderly population before and after IPTW are shown in Supplementary Table 6.

**Figure 3.**
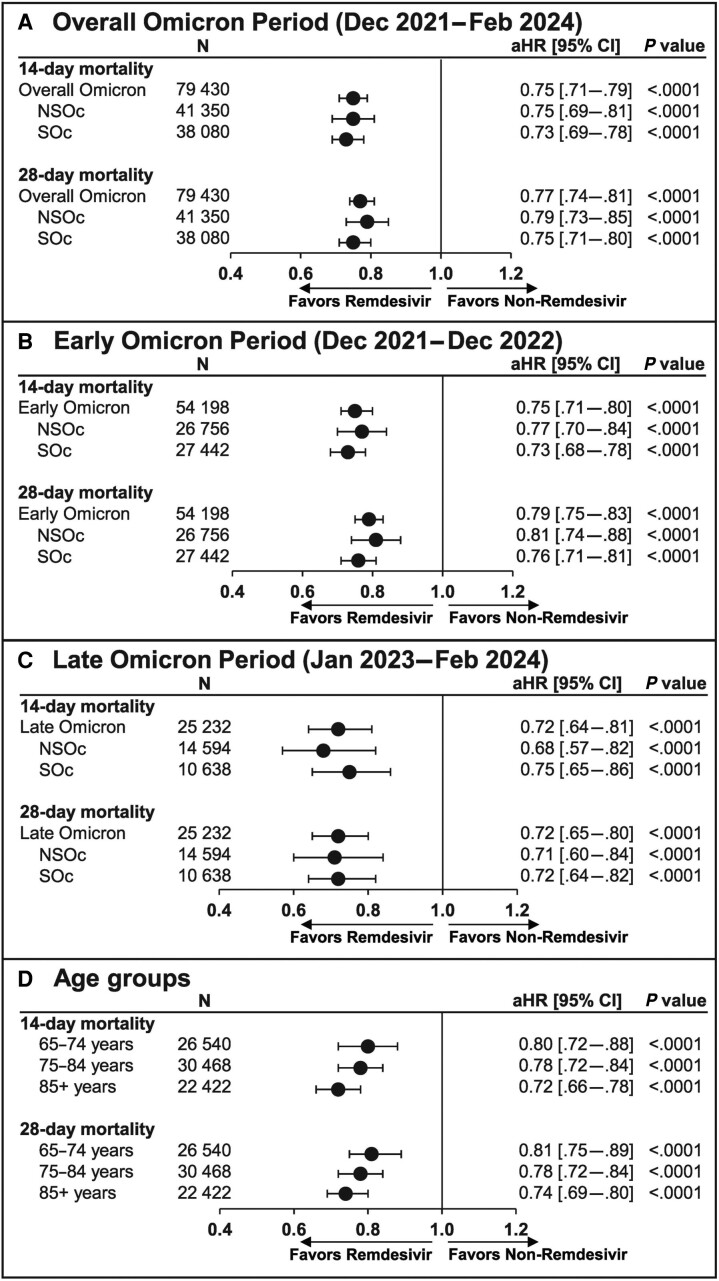
The 14- and 28-day mortality in elderly patients hospitalized for coronavirus disease 2019 (COVID-19) who received and did not receive remdesivir: propensity score matching. Cox proportional hazards model used to derive estimates adjusted for age, admission month, hospital ward upon admission (intensive care unit vs general ward), and time-varying treatment with other COVID-19 medications (baricitinib, tocilizumab, oral antivirals). (*A*), Overall Omicron period. (*B*), Early Omicron period. (*C*), Late Omicron period. (*D*), Age groups (65–74, 75–84, and ≥85 years). Abbreviations: aHR, adjusted hazard ratio; CI, confidence interval; NSOc, no supplemental oxygen charges; SOc, supplemental oxygen charges.

Unadjusted mortality risk was 8.1% vs 10.5% at 14 days and 10.5% vs 13.0% at 28 days for the remdesivir vs no-remdesivir groups, respectively. After adjusting for baseline and clinical covariates, remdesivir was associated with a significantly lower 14- and 28-day mortality rate compared with no remdesivir (aHR [95% CI]: 0.75 [.71–.79] and 0.77 [.74–.81], respectively; *P* < .0001; Figure 3*A*). These findings were consistent for the early (Figure 3*B*) and late Omicron periods (Figure 3*C*) and across age groups (65–74 years, 75–84 years, and ≥85 years) (Figure 3*D*).

For the NSOc subpopulation, unadjusted mortality risk for the entire Omicron period was 5.3% vs 6.7% at 14 days and 6.7% vs 8.0% at 28 days for the remdesivir vs no-remdesivir groups, respectively. After adjusting for baseline and clinical covariates, remdesivir was associated with a significantly lower 14- and 28-day mortality rate compared with no remdesivir (aHR [95% CI]: 0.75 [.69–.81] and 0.79 [.73–.85], respectively; *P* < .0001; Figure 3*A*). Additional data for the early and late Omicron periods are shown in Figures 3*B* and 3*C*, respectively.

For the SOc subpopulation, unadjusted mortality risk for the entire Omicron period was 11.2% vs 14.7% at 14 days and 14.6% vs 18.5% at 28 days for the remdesivir vs no-remdesivir groups, respectively. After adjusting for baseline and clinical covariates, remdesivir was associated with a significantly lower 14- and 28-day mortality rate compared with no remdesivir (aHR [95% CI]: 0.73 [.69–.78] and 0.75 [.71–.80], respectively; *P* < .0001; Figure 3*A*). Additional data for the early and late Omicron periods are shown in Figures 3*B* and 3*C*, respectively.

These findings were also consistent for the sensitivity analyses that used IPTW (Supplementary Table 7) and for the sensitivity analysis that compared remdesivir initiation in the first 2 days of admission vs no remdesivir initiation in the first 2 days of admission (Supplementary Table 8).

### COVID-19 Pneumonia Population

The COVID-19 pneumonia population included 112 683 patients of whom 68 507 (60.8%) initiated remdesivir in the first 2 days of hospitalization and 44 176 (39.2%) were not treated with remdesivir during the hospitalization (Figure 1). Before PSM, most patients in the remdesivir and no-remdesivir cohorts, respectively, were aged ≥65 years (67%, 70%) and had cardiovascular disease (86%, 88%). A lower proportion of patients who received remdesivir compared with those who did not receive remdesivir had renal disease (24%, 35%), while a higher proportion required supplemental oxygen (63%, 57%). Median duration of remdesivir use was 5.0 days [IQR Q1, Q3 4.0, 5.0). After 1:1 PSM, 36 385 patients who received remdesivir were matched to 36 385 patients who did not receive remdesivir during hospitalization. Characteristics were well balanced after PSM, with all covariates demonstrating an absolute standardized difference of <0.15 (Supplementary Table 9, Supplementary Figure 3). Baseline demographics and hospital characteristics of the COVID-19 pneumonia population before and after IPTW are shown in Supplementary Table 10.

**Figure 4.**
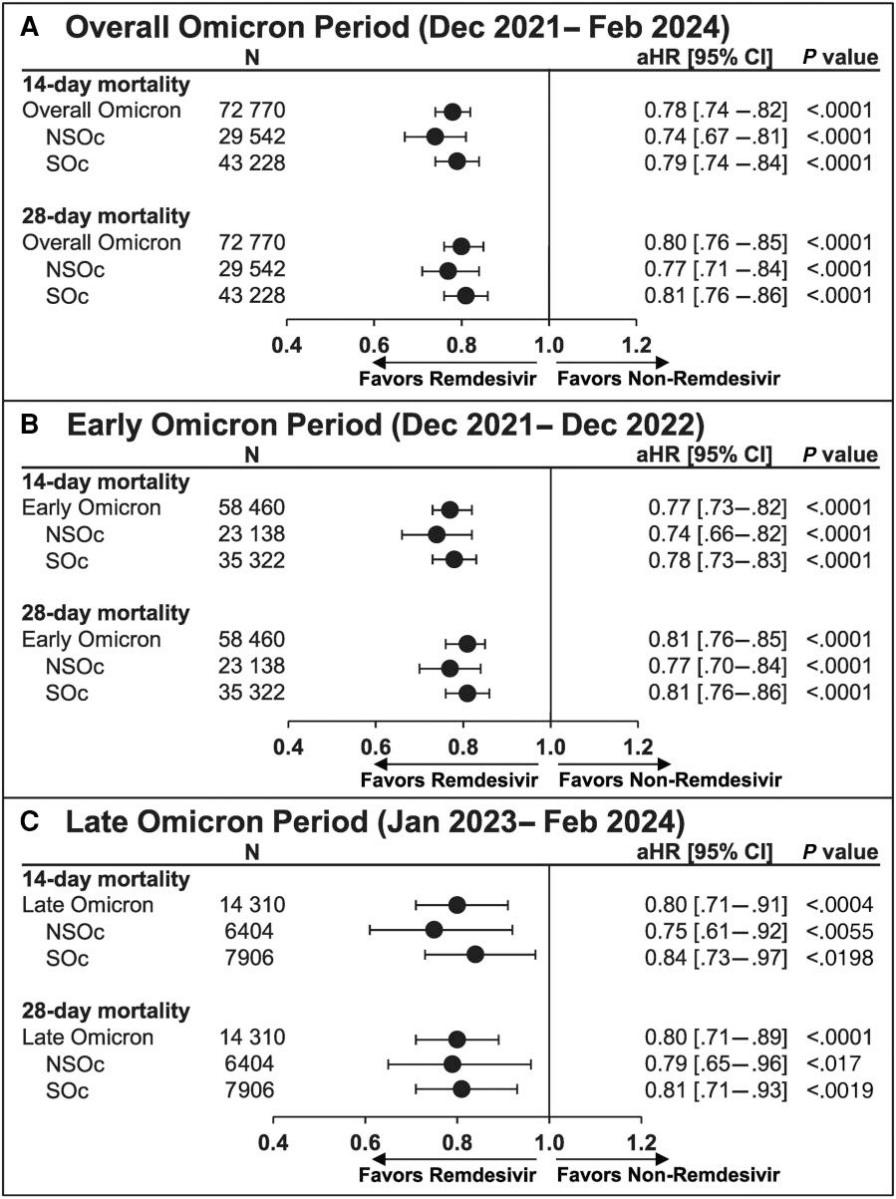
The 14- and 28-day mortality in patients hospitalized for coronavirus disease 2019 (COVID-19) pneumonia who received and did not receive remdesivir: propensity score matching. Cox proportional hazards model used to derive estimates adjusted for age, admission month, hospital ward upon admission (intensive care unit vs general ward), and time-varying treatment with other COVID-19 medications (baricitinib, tocilizumab, oral antivirals). (*A*), Overall Omicron period. (*B*), Early Omicron period. (*C*), Late Omicron period. Abbreviations: aHR, adjusted hazard ratio; CI, confidence interval; NSOc, no supplemental oxygen charges; SOc, supplemental oxygen charges.

Unadjusted mortality risk was 9.1% vs 11.3% at 14 days and 12.4% vs 14.9% at 28 days for the remdesivir vs no-remdesivir groups, respectively. After adjusting for baseline and clinical covariates, remdesivir was associated with a significantly lower 14- and 28-day mortality rate compared with no remdesivir (aHR [95% CI]: 0.78 [.74–.82] and 0.80 [.76–.85], respectively; *P* < .0001; Figure 4*A*). These findings were consistent for the early (Figure 4*B*) and late Omicron periods (Figure 4*C*).

For the NSOc subpopulation, unadjusted mortality risk for the entire Omicron period was 5.7% vs 7.4% at 14 days and 7.7% vs 9.4% at 28 days for the remdesivir vs no-remdesivir groups, respectively. After adjusting for baseline and clinical covariates, remdesivir was associated with a significantly lower 14- and 28-day mortality rate compared with no remdesivir (aHR [95% CI]: 0.74 [.67–.81] and 0.77 [.71–.84], respectively; *P* < .0001; Figure 4*C*). Additional data for the early and late Omicron periods are shown in Figures 4*B* and 4*C*, respectively.

For the SOc subpopulation, unadjusted mortality risk for the entire Omicron period was 11.4% vs 14.0% at 14 days and 15.6% vs 18.6% at 28 days for the remdesivir vs no-remdesivir groups, respectively. After adjusting for baseline and clinical covariates, remdesivir was associated with a significantly lower 14- and 28-day mortality rate compared with no remdesivir (aHR [95% CI]: 0.79 [.74–.84] and 0.81 [.76–.86], respectively; *P* < .0001; Figure 4*A*). Similar findings for the early and late Omicron periods are shown in Figures 4*B* and 4*C*, respectively.

These findings were also consistent for the sensitivity analyses that used IPTW (Supplementary Table 11) and for the sensitivity analysis that compared remdesivir initiation in the first 2 days of admission vs no remdesivir initiation in the first 2 days of admission (Supplementary Table 12).

## DISCUSSION

In 2023, there were more than 75 000 deaths in the US from COVID-19 and more than 25 000 deaths through 30 June 2024 [21]. In our matched study cohorts, the 28-day in-hospital mortality rate observed among all patients hospitalized for COVID-19 was 10.5%. Elderly patients (71% of patients in our sample) and patients with a secondary diagnosis of pneumonia due to COVID-19 (66% of patients in our sample) experienced a 28-day mortality risk of 11.8% and 13.6%, respectively. These data illustrate that COVID-19 remains a substantial and persistent clinical challenge and that patients hospitalized for COVID-19 continue to experience high rates of mortality, even as the pandemic has subsided.

Remdesivir has been available since the early phases of the pandemic [8]. Based on its proven efficacy and safety, it is included in the treatment guidelines for routine use in most patients hospitalized for COVID-19 [14, 15, 22, 23]. Although the effectiveness of remdesivir in the Omicron-dominant era has been confirmed in the database used in this study, treatment-associated adverse event reporting is not available and, hence, was not examined. However, remdesivir has been proven to be safe and well tolerated for the treatment of COVID-19 in hospitalized patients, as evidenced by prior research, including RCTs and real-world studies [8, 24]. Despite this, substantial number of patients continue to be hospitalized for COVID-19 in the United States today who are not receiving remdesivir. During the Omicron variant era, 45% of patients were not receiving remdesivir (Supplementary Table 2). Patients who did not receive remdesivir experienced significantly higher rates of mortality than those who did receive remdesivir according to adjusted analysis (21% higher 28-day mortality). This finding is consistent with the accumulated evidence from both RCTs and real-world evidence studies that consistently observed survival benefits with remdesivir [13]. The reasons are unclear why a substantial proportion of patients hospitalized with COVID-19 did not receive remdesivir. Of the 45% of patients who did not receive remdesivir on admission, less than 2% received any oral antiviral treatment; however, nearly 60% received a corticosteroid without any other known COVID-19 treatment, despite the recommendation against its use as monotherapy (specifically for dexamethasone) except for within a small subset of patients with the highest severity [15]. Additionally, about half of the patients without any oxygen supplementation at baseline received dexamethasone during the baseline period despite its proven harm for this subpopulation [25].

This study provides strong evidence that remdesivir continues to be associated with significant improved survival outcomes in routine clinical practice in the management of patients hospitalized for COVID-19 in the current endemic era, as had been observed during previous time periods [10–13, 16, 19, 26]. Our research is based on a large data source within which numerous real-world studies and comparative effectiveness assessments have been conducted, published, and used in clinical decision-making support [17]. To meet the need for contemporaneous evidence in the endemic era, a unique feature of our study is the comparison of the early and late Omicron periods. More importantly, the methods used in this research study represent rigorous approaches for real-world research, including a robust PSM methodology and secondary multivariate adjustment to account for between-group differences in factors including patient demographics, comorbidities, treatments (eg, corticosteroids, other antivirals), geography, and hospital-level characteristics across the geographically diverse patient population. Lower mortality rates following remdesivir therapy were observed regardless of the need for supplemental oxygen therapy and were consistent for the early and later Omicron periods. Our findings reconfirm the importance of initiating remdesivir therapy in adult patients hospitalized for COVID-19 during the Omicron period and bridge the gap between RCTs conducted during the earlier pandemic era and today's clinical setting in the endemic era. In the absence of expected guideline updates [15] and given the evolution of SARS-CoV-2 during this endemic period, our study offers current evidence to support and evolve clinical decision-making for managing today's COVID-19 patients. In the absence of new RCTs, hospitalists and internists who manage today's COVID-19 patients, among their many other duties, must rely on available guidelines and treatment protocols established based on real-world evidence, mostly from the pre-Omicron era. The infectious diseases and pulmonology specialist communities must continually and systematically assess the latest evidence and educate the broader hospitalist/internist community to eliminate any lingering or evolving gaps in our understanding of the appropriate care for today's COVID-19 patients.

As with any observational study, our research findings should be interpreted with caution. Residual confounding due to differences in measured and unmeasured factors can lead to imbalances between groups that can persist even after PSM and have an unknown influence on the observed findings. For example, providers’ subjective impressions of patients’ health status were neither captured in this database nor were some other important clinical factors such as vaccination status and rate of previous infections, which may have affected providers’ decisions to prescribe remdesivir or not. However, all patients included in this study were already hospitalized for COVID-19, reflecting a failed protection from prior immunity; hence, the bias introduced would be reduced. Clinical decision-making may be impacted as clinicians may be more likely to prescribe remdesivir to those who are unvaccinated, which would underestimate the benefit of remdesivir observed in this study. In addition, it could be expected that the PSM approach that led to the balancing of the measured variables in this study (specifically, age and key comorbidities) is likely to have (at least partially) balanced out unmeasured variables such as vaccination and prior infection as well. Misclassification of some important confounding variables is possible because variables based on billing and the ICD-10-CM coding may misclassify or underrepresent comorbid conditions, treatments, and procedures. Further, the no-remdesivir group could include patients who were not given remdesivir due to a potential historical contraindication for remdesivir administration prior to its current updated and expanded label [8]. Lastly, this database does not provide confirmatory testing and imaging findings, necessitating the use of proxy measures of disease severity, such as evidence of oxygen use. While it is likely that such factors (both known and unknown) are ultimately balanced between groups after PSM and multivariate adjustment, imbalances cannot be ruled out.

## CONCLUSIONS

Using a large sample of patients hospitalized for COVID-19 in the Omicron era that spanned 3 years, we found high ongoing mortality despite the end of the most severe phases of the pandemic. We also found a high proportion of hospitalized patients who did not receive antiviral therapy. When compared with those who were not treated, treatment with remdesivir was associated with a significantly lower mortality across broad patient subgroups, including the elderly and those with and without pneumonia, regardless of their need for supplemental oxygen. These results suggest that remdesivir treatment should be considered a standard of care for those hospitalized with COVID-19, even with widespread population-based immunity.

## Supplementary Data

Supplementary materials are available at *Clinical Infectious Diseases* online. Consisting of data provided by the authors to benefit the reader, the posted materials are not copyedited and are the sole responsibility of the authors, so questions or comments should be addressed to the corresponding author.

## Supplementary Material

ciae512_Supplementary_Data

## Notes

***Author Contributions.*** All authors contributed substantially to the manuscript and agreed to the final submitted version.

***Acknowledgments.*** Medical writing support was provided by Teresa Tartaglione, PharmD of Certara.

***Financial support***. This study was funded by Gilead Sciences, Inc.

***Supplement sponsorship.*** This article appears as part of the supplement “Harnessing Real-world Data to Inform Clinical Practice: Lessons From the Evolving COVID-19 Era,” sponsored by Gilead Sciences, Inc.

***Potential conflicts of interest.*** E. M. and M. B. are employees and stockholders of Gilead Sciences. A. C. and R. C. received funding and medical writing support from Gilead Sciences for conduct of this study. P. E. S. reports research grants from Gilead Sciences and ViiV and consulting fees from Gilead Sciences, Merck, and ViiV. P. L. reports payment or honoraria for lectures, presentations, speakers’ bureaus, manuscript writing, or educational events from AstraZeneca, Pfizer, and Moderna. Y. D. reports research grants from Entasis, payment or honoraria for lectures from Shionogi and BD and participation on advisory boards for Gilead Sciences, GSK, Moderna, Shionogi, MeijiSeika Pharma, Pfizer, AbbVie, all outside of the submitted work. A. N. A. served as principal investigator or co-principal investigator of clinical trials sponsored by the National Institutes of Health/National Institute of Allergy and Infectious Diseases, NeuroRx Pharma, Pulmotect, Blade Therapeutics, Novartis, Takeda, Humanigen, Eli Lilly, PTC Therapeutics, OctaPharma, Fulcrum Therapeutics, Alexion and as a speaker and/or consultant for Pfizer, Salix, Alexion, AstraZeneca, Bayer, Ferring, Seres, Spero, Eli Lilly, Novo Nordisk, Gilead Sciences, Renibus, GSK, Dexcom, Reprieve, HeartRite, and Aseptiscope, all outside the submitted work. N. A. reports consulting fees from Gilead Sciences; payment or honoraria for lectures, presentations, speakers’ bureaus, manuscript writing, or educational events from Gilead Sciences and BMJ; support for attending meetings and/or travel from Gilead Sciences; and stock or stock options from Atropos. V. M. reports consultancy fees from AstraZeneca, Actelion, Boehringer Ingelheim, Berlin-Chemie Menarini, BMS, Chiesi, Gilead Sciences, Lilly, MSD, Novartis, Richter, and Roche. M. K. reports research grants from Pfizer; payment or honoraria for lectures from AstraZeneca, Berlin-Chemie, Boehringer Ingelheim, Gilead Sciences, GSK, Insmed, Pfizer; and participation on advisory boards for AstraZeneca, Sanofi, GSK, MSD, and Insmed, all outside of the submitted work.

***Ethics approval and consent to participate.*** This study did not require ethics approval and informed consent. The analysis of data from the Premier Healthcare Database (US PINC AI Healthcare Database) was conducted under an exemption from the Institutional Review Board oversight for US-based studies using de-identified healthcare records that are not re-identifiable.

***Availability of data and methods.*** The data that support this study's findings are available from Premier, Inc (https://www.premierinc.com/). Restrictions apply to the availability of these data, which were used under license for this study.
