# Supplementary material for: Management of Vulnerable Patients Hospitalized for COVID-19 With Remdesivir: A Retrospective Comparative Effectiveness Study of Mortality in US Hospitals
Source: Clin Infect Dis. 2024 Oct 19;79(Suppl 4):S137–48. doi: 10.1093/cid/ciae512 (PMC11638768; doi:10.1093/cid/ciae512)
Supplement: ciae512_Supplementary_Data [file ciae512_supplementary_data.docx]

**Management of Vulnerable Patients Hospitalized for COVID-19 With Remdesivir: A Retrospective Comparative Effectiveness Study of Mortality in US Hospitals**

Essy Mozaffari,^1^ Aastha Chandak,^2^ Mark Berry,^1^ Paul E. Sax,^3^ Paul Loubet,^4^ Yohei Doi,^5,6^ Alpesh N. Amin,^7^ Neera Ahuja,^8^ Veronika Müller,^9^ Roman Casciano,^2^ Martin Kolditz^10^

*^1^Medical Affairs, Gilead Sciences, Foster City, California, USA*

*^2^Evidence and Access, Certara, New York City, New York, USA*

*^3^Division of Infectious Diseases, Brigham and Women’s Hospital, Boston, Massachusetts, USA*

*^4^Department of Infectious and Tropical Diseases, Centre Hospitalier Universitaire de Nimes, Nimes, France*

*^5^Departments of Microbiology and Infectious Disease, Fujita Health University School of Medicine, Toyoake, Japan*

*^6^Division of Infectious Diseases, University of Pittsburgh School of Medicine, Pittsburgh, Pennsylvania, USA*

*^7^Department of Medicine, School of Medicine, University of California Irvine, Irvine, California, USA*

*^8^Department of Internal Medicine, Stanford University School of Medicine, Stanford, California, USA*

*^9^Department of Pulmonology, Semmelweis University, Budapest, Hungary*

*^10^Medical Department I, University Hospital Carl Gustav Carus of TU Dresden, Dresden, Saxony, Germany*

**Corresponding Author**:

Prof. Dr. med. Martin Kolditz

Medical Department I, University Hospital Carl Gustav Carus of TU Dresden, Dresden, Saxony, Germany

Tel. +49 (0)351 458-3417

Fax +49 (0)351 458-5765

[Martin.Kolditz@ukdd.de](mailto:Martin.Kolditz@ukdd.de)

# SUPPLEMENTARY TABLES AND FIGURES

# Supplementary Table 1. Definitions of Key Study Variables

| Key Study Variables | | Definitions |
| --- | --- | --- |
| Remdesivir treatment |  | Billing charges for treatment: Remdesivir;  ICD-10 procedure codes: XW033E5, XW043E5 |
| Key Comorbidities | Obesity | ICD-10-CM diagnosis codes: E66, Z6825-Z6845 |
|  | COPD | ICD-10-CM diagnosis codes: I278, I279, J40, J41, J42, J43, J44, J45, J46, J47, J60, J61, J62, J63, J64, J65, J66, J67, J684, J701, J703 |
|  | Cardiovascular disease (including hypertension) | ICD-10-CM diagnosis codes: I00-I99 |
|  | Diabetes | ICD-10-CM diagnosis codes: E10-E14 |
|  | Renal disease | ICD-10-CM diagnosis codes: I120, I131, N032, N033, N034, N035, N036, N037, N052, N053, N054, N055, N056, N057, N18, N19, N250, Z490, Z491, Z492, Z940, Z992 |
|  | Cancer | ICD-10-CM diagnosis codes: C00-C96 |
|  | Immunocompromising condition | ICD-10-CM code for cancer (C00-C96), transplant (Z94.x), hematologic malignancies (C81.x, C82.x, C83.x, C84.x, C85.x, C88.x, C90.x, C91.x, C92.x, C93.x, C94.x, C95.x, C96.x), primary immunodeficiencies (D80.x, D81.x, D82.x, D83.x, D84.x, G11.3, E70.330, D71.x, D70.x), asplenia (Q89.01, Z90.81), toxic effects of antineoplastics (T45.1x), bone marrow failure/aplastic anemia (D61.x), severe combined immunodeficiencies (D80.x, D81.x, D82.x, D83.x, D84.x, D86.x, D89.0, D89.1, D89.2, D89.3, D89.4x, D89.81, D89.82, D89.89, D89.9), HIV (B20), patients with chronic graft-versus-host disease or who are taking immunosuppressive medications for another indication (Z89.8x, Z79.52, Z79.61, Z79.62x, Z79.63x, Z79.64, Z79.69, Z79.810, Z79.811, Z79.818) |
| Supplemental oxygen requirements | IMV | Billing charges for devices: invasive mechanical ventilation, tracheostomy, endotracheal tube intubation, |
|  | ECMO | Billing charges for devices: extracorporeal membrane oxygenation |
|  | HFO/NIV | Billing charges for devices: negative-pressure ventilation, positive-pressure ventilation, CPAP, BiPAP, high flow system via nasal cannula, venturi face mask, rebreather, non-rebreather mask, positive expiratory pressure |
|  | LFO | Billing charges for devices/oxygen supply: simple face mask, oxygen pendant, low-flow system via nasal cannula, oxygen supply |
|  | NSOc | No billing charges for IMV, ECMO, HFO/NIV, or LFO at baseline |
| Admitting Diagnosis | Sepsis | ICD-10-CM diagnosis codes: A021, A327, A40, A41, A427, A5486, B377, R6520, R6521, T8144X, |
|  | Pneumonia | ICD-10-CM diagnosis codes: J12, J13, J14, J15, J16, J17, J18, A481, B250, A3701, A3711, A3781, A3791, A221, B440, B7781, J1000, J1001, J1008, J1100, J1108 |
| Baseline Medications | Anticoagulants | Billing charges for treatment at baseline: apixaban, argatroban, desirudin, lepirudin, dabigatran, danaparoid, edoxaban, tinzaparin, heparin (excluding use of heparin flush), ardeparin, bivalirudin |
|  | Corticosteroids | Billing charges for treatment at baseline: prednisone, prednisolone, methylprednisolone, hydrocortisone, dexamethasone |
|  | Convalescent plasma | Billing charges for treatment at baseline: convalescent plasma; ICD-10 procedure codes: XW13325, XW14325 |
|  | Tocilizumab | Billing charges for treatment at baseline: tocilizumab; ICD-10 procedure codes: XW033H5, XW043H5 |
|  | Baricitinib | Billing charges for treatment at baseline: Baricitinib; ICD-10 procedure codes: XW0DXM6, XW0H7M6, XW0G7M6 |
|  | Oral antivirals | Billing charges for treatment at baseline: nirmatrelvir ritonavir, molnupiravir |
| Abbreviations: BiPAP, bilevel positive airway pressure; CPAP, continuous positive airway pressure; COPD, chronic obstructive pulmonary disease; HFO/NIV, high flow oxygen/non-invasive ventilation; ICD-10-CM, International Classification of Diseases, 10th Revision (Clinical Modification); LFO, low flow oxygen; IMV, invasive mechanical ventilation; ECMO, extracorporeal membrane oxygenation; NSOc, no supplemental oxygen charges | | |

# Supplementary Table 2. Demographics of Adults Hospitalized for COVID-19 During December 2021-February 2024 Before and After Inverse Probability of Treatment Weighting

| **Characteristic** | | **Before IPTW** | | | **After IPTW** | | |
| --- | --- | --- | --- | --- | --- | --- | --- |
|  |  | **No Remdesivir**  **n = 75,836** | **Remdesivir**  **n = 94,129** | **SMD** | **No Remdesivir** | **Remdesivir** | **SMD** |
| Age group, years | 18–49 | 6,769 (8.9%) | 8,689 (9.2%) | 0.05 | 9.00% | 9.00% | 0.00 |
|  | 50–64 | 14,335 (18.9%) | 19,642 (20.9%) |  | 20.00% | 20.00% |  |
|  | ≥65 | 54,732 (72.2%) | 65,798 (69.9%) |  | 70.90% | 71.10% |  |
| Gender | Female | 38,980 (51.4%) | 47,961 (51.0%) | 0.01 | 51.10% | 51.10% | 0.00 |
| Race | White | 56,934 (75.1%) | 72,221 (76.7%) | 0.09 | 76.00% | 76.00% | 0.00 |
|  | Black | 12,148 (16.0%) | 12,230 (13.0%) |  | 14.30% | 14.30% |  |
|  | Asian | 1,383 (1.8%) | 2,266 (2.4%) |  | 2.20% | 2.20% |  |
|  | Other | 5,371 (7.1%) | 7,412 (7.9%) |  | 7.60% | 7.50% |  |
| Ethnicity | Hispanic | 5,931 (7.8%) | 10,103 (10.7%) | 0.11 | 9.40% | 9.40% | 0.00 |
|  | Non-Hispanic | 64,361 (84.9%) | 78,574 (83.5%) |  | 84.20% | 84.20% |  |
|  | Unknown | 5,544 (7.3%) | 5,452 (5.8%) |  | 6.40% | 6.40% |  |
| Primary payor | Commercial | 9,662 (12.7%) | 14,670 (15.6%) | 0.13 | 14.30% | 14.30% | 0.00 |
|  | Medicare | 55,698 (73.4%) | 66,690 (70.8%) |  | 72.00% | 72.20% |  |
|  | Medicaid | 6,388 (8.4%) | 8,177 (8.7%) |  | 8.60% | 8.50% |  |
|  | Other | 4,088 (5.4%) | 4,592 (4.9%) |  | 5.10% | 5.10% |  |
| Admission source | Transfer from SNF or ICF | 2,651 (3.5%) | 3,589 (3.8%) | 0.02 | 3.70% | 3.70% | 0.00 |
| Hospital size, number of beds | <100 | 6,136 (8.1%) | 7,301 (7.8%) | 0.10 | 8.10% | 8.00% | 0.00 |
|  | 100–199 | 12,194 (16.1%) | 16,260 (17.3%) |  | 16.80% | 16.80% |  |
|  | 200–299 | 16,267 (21.5%) | 19,030 (20.2%) |  | 20.70% | 21.00% |  |
|  | 300–399 | 14,330 (18.9%) | 15,263 (16.2%) |  | 17.00% | 17.00% |  |
|  | 400–499 | 8,532 (11.3%) | 10,267 (10.9%) |  | 11.20% | 11.20% |  |
|  | 500+ | 18,377 (24.2%) | 26,008 (27.6%) |  | 26.10% | 26.00% |  |
| Hospital location | Urban | 66,070 (87.1%) | 83,163 (88.4%) | 0.04 | 87.70% | 87.80% | 0.00 |
|  | Rural | 9,766 (12.9%) | 10,966 (11.6%) |  | 12.30% | 12.20% |  |
| Teaching hospital | | 31,286 (41.3%) | 40,685 (43.2%) | 0.04 | 42.3% | 42.4% | 0.00 |
| Region | Midwest | 19,227 (25.4%) | 22,369 (23.8%) | 0.15 | 24.8% | 24.7% | 0.00 |
|  | Northeast | 8,727 (11.5%) | 15,532 (16.5%) |  | 14.3% | 14.2% |  |
|  | South | 39,694 (52.3%) | 44,582 (47.4%) |  | 49.1% | 49.2% |  |
|  | West | 8,188 (10.8%) | 11,646 (12.4%) |  | 11.8% | 11.8% |  |
| Comorbid conditions | Obesity | 20,398 (26.9%) | 28,023 (29.8%) | 0.06 | 28.5% | 28.6% | 0.00 |
|  | COPD | 25,221 (33.3%) | 36,133 (38.4%) | 0.11 | 36.3% | 36.3% | 0.00 |
|  | Cardiovascular disease | 67,117 (88.5%) | 81,565 (86.7%) | −0.06 | 87.6% | 87.6% | 0.00 |
|  | Diabetes | 30,277 (39.9%) | 36,340 (38.6%) | −0.03 | 39.2% | 39.3% | 0.00 |
|  | Renal disease | 25,492 (33.6%) | 23,827 (25.3%) | −0.18 | 29.2% | 29.3% | 0.00 |
|  | Immunocompromising condition | 12,236 (16.1%) | 16,730 (17.8%) | 0.04 | 17.3% | 17.3% | 0.00 |
|  | Cancer | 5,125 (6.8%) | 7,163 (7.6%) | 0.03 | 7.3% | 7.3% | 0.00 |
| Hospital ward on admission | General ward | 63,560 (83.8%) | 75,932 (80.7%) | 0.08 | 82.1% | 82.0% | 0.00 |
|  | Intensive care unit/step down unit | 12,276 (16.2%) | 18,197 (19.3%) |  | 17.9% | 18.0% |  |
| Key diagnosis on admission | Sepsis | 365 (0.5%) | 342 (0.4%) | −0.02 | 0.4% | 0.4% | 0.00 |
|  | Pneumonia | 4,622 (6.1%) | 6,036 (6.4%) | 0.01 | 6.2% | 6.2% | 0.00 |
| Other COVID-19 treatments at baseline | Anticoagulants | 53,852 (71.0%) | 73,580 (78.2%) | 0.16 | 75.0% | 74.8% | 0.00 |
|  | Convalescent plasma | 30 (0.0%) | 105 (0.1%) | 0.54 | 0.1% | 0.1% | 0.00 |
|  | Corticosteroids | 47,557 (62.7%) | 80,504 (85.5%) | 0.03 | 75.6% | 75.7% | 0.00 |
|  | Baricitinib | 4,103 (5.4%) | 5,005 (5.3%) | 0.08 | 5.4% | 5.5% | 0.00 |
|  | Tocilizumab | 1,863 (2.5%) | 3,543 (3.8%) | 0.00 | 3.3% | 3.2% | 0.00 |
|  | Oral antivirals | 1,306 (1.7%) | 204 (0.2%) | −0.15 | 0.7% | 0.7% | 0.00 |
| Baseline supplemental oxygen requirements | NSOc | 41,894 (55.2%) | 41,013 (43.6%) | 0.27 | 2.9% | 2.9% | 0.00 |
|  | LFO | 20,794 (27.4%) | 31,808 (33.8%) |  | 17.4% | 17.4% |  |
|  | HFO/NIV | 10,598 (14.0%) | 18,887 (20.1%) |  | 30.9% | 31.1% |  |
|  | IMV | 2,550 (3.4%) | 2,421 (2.6%) |  | 48.8% | 48.6% |  |
| Omicron period | Early (Dec 2021-Dec 2022) | 54,372 (71.7%) | 66,484 (70.6%) | 0.02 | 70.9% | 70.9% | 0.00 |
|  | Late (Jan 2023-Feb 2024) | 21,464 (28.3%) | 27,645 (29.4%) |  | 29.1% | 29.1% |  |
| Abbreviations: COPD, chronic obstructive pulmonary disease; COVID-19, coronavirus disease 2019; HFO/NIV, high flow oxygen/non-invasive ventilation; ICF, intermediate care facility; IMV, invasive mechanical ventilation; IPTW, inverse probability of treatment weighting; LFO, low flow oxygen; NSOc, no supplemental oxygen charges; PSM, propensity score matching; SMD, standardized mean difference; SNF, skilled nursing facility | | | | | | | |

# Supplementary Table 3. 14- and 28-day Mortality in Adults Hospitalized for COVID-19 Receiving Remdesivir vs no Remdesivir by Supplemental Oxygen Requirements: Inverse Probability of Treatment Weighting

|  | **aHR [95% CI]** | **P-value** |
| --- | --- | --- |
| **14-day mortality** |  |  |
| Overall Omicron | 0.76 [0.72 – 0.79] | <.0001 |
| NSOc | 0.72 [0.67 – 0.77] | <.0001 |
| SOc | 0.76 [0.72 – 0.80] | <.0001 |
| **28-day mortality** |  |  |
| Overall Omicron | 0.78 [0.75 – 0.82] | <.0001 |
| NSOc | 0.76 [0.71 – 0.81] | <.0001 |
| SOc | 0.78 [0.74 – 0.82] | <.0001 |

Cox Proportional Hazards model used to derive estimates adjusted for age, admission month, hospital ward on admission (ICU vs general ward), and time-varying treatment with other COVID-19 medications (baricitinib, tocilizumab, oral antivirals).

Abbreviations: aHR, adjusted hazard ratio; CI, confidence interval; COVID-19, coronavirus disease 2019; NSOc, no supplemental oxygen charges; SOc, supplemental oxygen charges

# Supplementary Table 4. 14- and 28-day Mortality in Adults Hospitalized for COVID-19 Receiving Remdesivir in the First Two Days vs no Remdesivir in the First Two Days by Supplemental Oxygen Requirements: Propensity Score Matching

|  | **N** | **aHR [95% CI]** | **P-value** |
| --- | --- | --- | --- |
| **14-day mortality** |  |  |  |
| Overall Omicron | 120,174 | 0.76 [0.72 – 0.79] | <.0001 |
| NSOc | 58,688 | 0.73 [0.68 – 0.78] | <.0001 |
| SOc | 61,486 | 0.76 [0.72 – 0.81] | <.0001 |
| **28-day mortality** |  |  |  |
| Overall Omicron | 120,174 | 0.77 [0.73 – 0.80] | <.0001 |
| NSOc | 58,688 | 0.74 [0.70 – 0.79] | <.0001 |
| SOc | 61,486 | 0.77 [0.73 – 0.81] | <.0001 |

Cox Proportional Hazards model used to derive estimates adjusted for age, admission month, hospital ward on admission (ICU vs general ward), and time-varying treatment with other COVID-19 medications (baricitinib, tocilizumab, oral antivirals).

Abbreviations: aHR, adjusted hazard ratio; CI, confidence interval; COVID-19, coronavirus disease 2019; NSOc, no supplemental oxygen charges; SOc, supplemental oxygen charges

# Supplementary Table 5. Demographics of Elderly Patients Hospitalized for COVID-19 During December 2021-February 2024 Before and After Propensity Score Matching

| **Characteristic** | | **Before PSM** | | | **After PSM** | | |
| --- | --- | --- | --- | --- | --- | --- | --- |
|  |  | **No Remdesivir**  **n = 54,732** | **Remdesivir**  **n = 65,798** | **SMD** | **No Remdesivir**  **n = 39,715** | **Remdesivir**  **n = 39,715** | **SMD** |
| Age group, years | 65–74 | 17,601 (32.2%) | 22,314 (33.9%) | 0.05 | 13,270 (33.4%) | 13,270 (33.4%) | 0.00 |
|  | 85–84 | 20,932 (38.2%) | 25,034 (38.0%) |  | 15,234 (38.4%) | 15,234 (38.4%) |  |
|  | ≥85 | 16,199 (29.6%) | 18,450 (28.0%) |  | 11,211 (28.2%) | 11,211 (28.2%) |  |
| Gender | Female | 28,637 (52.3%) | 34,202 (52.0%) | 0.01 | 20,676 (52.1%) | 20,701 (52.1%) | 0.00 |
| Race | White | 43,171 (78.9%) | 53,094 (80.7%) | 0.11 | 31,892 (80.3%) | 31,935 (80.4%) | 0.00 |
|  | Black | 7,114 (13.0%) | 6,662 (10.1%) |  | 4,533 (11.4%) | 4,463 (11.2%) |  |
|  | Asian | 1,075 (2.0%) | 1,749 (2.7%) |  | 849 (2.1%) | 838 (2.1%) |  |
|  | Other | 3,372 (6.2%) | 4,293 (6.5%) |  | 2,441 (6.1%) | 2,479 (6.2%) |  |
| Ethnicity | Hispanic | 3,487 (6.4%) | 6,059 (9.2%) | 0.13 | 2,740 (6.9%) | 2,610 (6.6%) | 0.00 |
|  | Non-Hispanic | 47,243 (86.3%) | 56,019 (85.1%) |  | 34,486 (86.8%) | 34,591 (87.1%) |  |
|  | Unknown | 4,002 (7.3%) | 3,720 (5.7%) |  | 2,489 (6.3%) | 2,514 (6.3%) |  |
| Primary payor | Commercial | 2,145 (3.9%) | 3,143 (4.8%) | 0.11 | 1,691 (4.3%) | 1,667 (4.2%) | 0.00 |
|  | Medicare | 50,139 (91.6%) | 59,822 (90.9%) |  | 36,343 (91.5%) | 36,355 (91.5%) |  |
|  | Medicaid | 754 (1.4%) | 1,055 (1.6%) |  | 560 (1.4%) | 547 (1.4%) |  |
|  | Other | 1,694 (3.1%) | 1,778 (2.7%) |  | 1,121 (2.8%) | 1,146 (2.9%) |  |
| Admission source | Transfer from SNF or ICF | 2,379 (4.3%) | 3,204 (4.9%) | 0.02 | 1,795 (4.5%) | 1,768 (4.5%) | 0.00 |
| Hospital size, number of beds | <100 | 4,638 (8.5%) | 5,103 (7.8%) | 0.12 | 3,310 (8.3%) | 3,313 (8.3%) | 0.04 |
|  | 100–199 | 8,996 (16.4%) | 11,387 (17.3%) |  | 6,597 (16.6%) | 6,594 (16.6%) |  |
|  | 200–299 | 11,923 (21.8%) | 13,336 (20.3%) |  | 8,634 (21.7%) | 8,507 (21.4%) |  |
|  | 300–399 | 10,459 (19.1%) | 10,680 (16.2%) |  | 7,184 (18.1%) | 7,106 (17.9%) |  |
|  | 400–499 | 6,194 (11.3%) | 7,330 (11.1%) |  | 4,560 (11.5%) | 4,765 (12.0%) |  |
|  | 500+ | 12,522 (22.9%) | 17,962 (27.3%) |  | 9,430 (23.7%) | 9,430 (23.7%) |  |
| Hospital location | Urban | 47,394 (86.6%) | 58,284 (88.6%) | 0.06 | 34,678 (87.3%) | 34,661 (87.3%) | 0.00 |
|  | Rural | 7,338 (13.4%) | 7,514 (11.4%) |  | 5,037 (12.7%) | 5,054 (12.7%) |  |
| Teaching hospital | | 21,856 (39.9%) | 28,340 (43.1%) | 0.06 | 15,951 (40.2%) | 15,996 (40.3%) | 0.00 |
| Region | Midwest | 13,896 (25.4%) | 15,341 (23.3%) | 0.17 | 10,031 (25.3%) | 9,845 (24.8%) | 0.00 |
|  | Northeast | 6,501 (11.9%) | 11,290 (17.2%) |  | 5,181 (13.0%) | 5,343 (13.5%) |  |
|  | South | 28,479 (52.0%) | 30,967 (47.1%) |  | 19,905 (50.1%) | 19,938 (50.2%) |  |
|  | West | 5,856 (10.7%) | 8,200 (12.5%) |  | 4,598 (11.6%) | 4,589 (11.6%) |  |
| Comorbid conditions | Obesity | 12,014 (22.0%) | 15,079 (22.9%) | 0.02 | 9,052 (22.8%) | 9,059 (22.8%) | 0.00 |
|  | COPD | 18,666 (34.1%) | 26,088 (39.6%) | 0.12 | 14,842 (37.4%) | 14,802 (37.3%) | 0.00 |
|  | Cardiovascular disease | 51,356 (93.8%) | 61,153 (92.9%) | −0.04 | 37,111 (93.4%) | 37,121 (93.5%) | 0.00 |
|  | Diabetes | 22,245 (40.6%) | 25,619 (38.9%) | −0.03 | 15,882 (40.0%) | 15,870 (40.0%) | 0.00 |
|  | Renal disease | 20,695 (37.8%) | 19,549 (29.7%) | −0.17 | 13,970 (35.2%) | 13,732 (34.6%) | −0.01 |
|  | Immunocompromising condition | 8,955 (16.4%) | 12,056 (18.3%) | 0.05 | 6,910 (17.4%) | 6,900 (17.4%) | 0.00 |
|  | Cancer | 4,136 (7.6%) | 5,679 (8.6%) | 0.04 | 3,158 (8.0%) | 3,167 (8.0%) | 0.00 |
| Hospital ward on admission | General ward | 46,665 (85.3%) | 53,525 (81.3%) | 0.11 | 33,507 (84.4%) | 33,605 (84.6%) | −0.01 |
|  | Intensive care unit/step down unit | 8,067 (14.7%) | 12,273 (18.7%) |  | 6,208 (15.6%) | 6,110 (15.4%) |  |
| Key diagnosis on admission | Sepsis | 258 (0.5%) | 241 (0.4%) | −0.02 | 168 (0.4%) | 179 (0.5%) | 0.00 |
|  | Pneumonia | 3,253 (5.9%) | 4,085 (6.2%) | 0.01 | 2,453 (6.2%) | 2,447 (6.2%) | 0.00 |
| Other COVID-19 treatments at baseline | Anticoagulants | 38,550 (70.4%) | 50,268 (76.4%) | 0.14 | 29,195 (73.5%) | 29,347 (73.9%) | 0.00 |
|  | Convalescent plasma | 17 (0.0%) | 49 (0.1%) | 0.53 | 16 (0.0%) | 12 (0.0%) | 0.00 |
|  | Corticosteroids | 33,342 (60.9%) | 55,041 (83.7%) | 0.02 | 30,095 (75.8%) | 30,107 (75.8%) | -0.01 |
|  | Baricitinib | 2,359 (4.3%) | 2,511 (3.8%) | 0.06 | 1,770 (4.5%) | 1,754 (4.4%) | 0.00 |
|  | Tocilizumab | 1,034 (1.9%) | 1,804 (2.7%) | −0.03 | 903 (2.3%) | 922 (2.3%) | 0.00 |
|  | Oral antivirals | 1,090 (2.0%) | 165 (0.3%) | −0.17 | 119 (0.3%) | 123 (0.3%) | 0.00 |
| Baseline supplemental oxygen requirements | NSOc | 31,151 (56.9%) | 30,268 (46.0%) | 0.23 | 20,675 (52.1%) | 20,675 (52.1%) | 0.00 |
|  | LFO | 15,191 (27.8%) | 22,122 (33.6%) |  | 12,488 (31.4%) | 12,488 (31.4%) |  |
|  | HFO/NIV | 6,971 (12.7%) | 12,100 (18.4%) |  | 5,820 (14.7%) | 5,820 (14.7%) |  |
|  | IMV | 1,419 (2.6%) | 1,308 (2.0%) |  | 732 (1.8%) | 732 (1.8%) |  |
| Omicron period | Early (Dec 2021-Dec 2022) | 37,298 (68.1%) | 43,244 (65.7%) | 0.05 | 27,099 (68.2%) | 27,099 (68.2%) | 0.00 |
|  | Late (Jan 2023-Feb 2024) | 17,434 (31.9%) | 22,554 (34.3%) |  | 12,616 (31.8%) | 12,616 (31.8%) |  |
| Abbreviations: COPD, chronic obstructive pulmonary disease; COVID-19, coronavirus disease 2019; HFO/NIV, high flow oxygen/non-invasive ventilation; ICF, intermediate care facility; IMV, invasive mechanical ventilation; LFO, low flow oxygen; NSOc, no supplemental oxygen charges; PSM, propensity score matching; SMD, standardized mean difference; SNF, skilled nursing facility | | | | | | | |

# Supplementary Table 6. Demographics of Elderly Patients Hospitalized for COVID-19 During December 2021-February 2024 Before and After Inverse Probability of Treatment Weighting

| **Characteristic** | | **Before IPTW** | | | **After IPTW** | | |
| --- | --- | --- | --- | --- | --- | --- | --- |
|  |  | **No Remdesivir**  **n =54,732** | **Remdesivir**  **n = 65,798** | **SMD** | **No Remdesivir** | **Remdesivir** | **SMD** |
| Age group, years | 65–74 | 17,601 (32.2%) | 22,314 (33.9%) | 0.05 | 33.2% | 33.2% | 0.00 |
|  | 75–84 | 20,932 (38.2%) | 25,034 (38.0%) |  | 38.2% | 38.1% |  |
|  | ≥85 | 16,199 (29.6%) | 18,450 (28.0%) |  | 28.6% | 28.7% |  |
| Gender | Female | 28,637 (52.3%) | 34,202 (52.0%) | −0.01 | 52.0% | 52.0% | 0.00 |
| Race | White | 43,171 (78.9%) | 53,094 (80.7%) | 0.11 | 79.8% | 79.8% | 0.00 |
|  | Black | 7,114 (13.0%) | 6,662 (10.1%) |  | 11.4% | 11.4% |  |
|  | Asian | 1,075 (2.0%) | 1,749 (2.7%) |  | 2.4% | 2.4% |  |
|  | Other | 3,372 (6.2%) | 4,293 (6.5%) |  | 6.5% | 6.4% |  |
| Ethnicity | Hispanic | 3,487 (6.4%) | 6,059 (9.2%) | 0.13 | 7.7% | 7.9% | 0.00 |
|  | Non-Hispanic | 47,243 (86.3%) | 56,019 (85.1%) |  | 85.9% | 85.7% |  |
|  | Unknown | 4,002 (7.3%) | 3,720 (5.7%) |  | 6.4% | 6.3% |  |
| Primary payor | Commercial | 2,145 (3.9%) | 3,143 (4.8%) | 0.11 | 4.3% | 4.4% | 0.10 |
|  | Medicare | 50,139 (91.6%) | 59,822 (90.9%) |  | 91.3% | 91.2% |  |
|  | Medicaid | 754 (1.4%) | 1,055 (1.6%) |  | 1.5% | 1.5% |  |
|  | Other | 1,694 (3.1%) | 1,778 (2.7%) |  | 2.9% | 2.9% |  |
| Admission source | Transfer from SNF or ICF | 2,379 (4.3%) | 3,204 (4.9%) | 0.02 | 4.7% | 4.6% | 0.00 |
| Hospital size, number of beds | <100 | 4,638 (8.5%) | 5,103 (7.8%) | 0.12 | 8.2% | 8.2% | 0.00 |
|  | 100–199 | 8,996 (16.4%) | 11,387 (17.3%) |  | 16.9% | 17.0% |  |
|  | 200–299 | 11,923 (21.8%) | 13,336 (20.3%) |  | 21.0% | 21.2% |  |
|  | 300–399 | 10,459 (19.1%) | 10,680 (16.2%) |  | 17.2% | 17.2% |  |
|  | 400–499 | 6,194 (11.3%) | 7,330 (11.1%) |  | 11.4% | 11.3% |  |
|  | 500+ | 12,522 (22.9%) | 17,962 (27.3%) |  | 25.3% | 25.2% |  |
| Hospital location | Urban | 47,394 (86.6%) | 58,284 (88.6%) | 0.06 | 87.6% | 87.7% | 0.00 |
|  | Rural | 7,338 (13.4%) | 7,514 (11.4%) |  | 12.4% | 12.3% |  |
| Teaching hospital | | 21,856 (39.9%) | 28,340 (43.1%) | 0.06 | 41.50% | 41.6% | 0.00 |
| Region | Midwest | 13,896 (25.4%) | 15,341 (23.3%) | 0.17 | 24.6% | 24.50% | 0.00 |
|  | Northeast | 6,501 (11.9%) | 11,290 (17.2%) |  | 14.8% | 14.7% |  |
|  | South | 28,479 (52.0%) | 30,967 (47.1%) |  | 48.8% | 48.9% |  |
|  | West | 5,856 (10.7%) | 8,200 (12.5%) |  | 11.8% | 11.8% |  |
| Comorbid conditions | Obesity | 12,014 (22.0%) | 15,079 (22.9%) | 0.02 | 22.5% | 22.5% | 0.00 |
|  | COPD | 18,666 (34.1%) | 26,088 (39.6%) | 0.12 | 37.4% | 37.4% | 0.00 |
|  | Cardiovascular disease | 51,356 (93.8%) | 61,153 (92.9%) | −0.04 | 93.4% | 93.4% | 0.00 |
|  | Diabetes | 22,245 (40.6%) | 25,619 (38.9%) | −0.03 | 39.8% | 39.8% | 0.00 |
|  | Renal disease | 20,695 (37.8%) | 19,549 (29.7%) | −0.17 | 33.7% | 33.7% | 0.00 |
|  | Immunocompromising condition | 8,955 (16.4%) | 12,056 (18.3%) | 0.05 | 17.7% | 17.7% | 0.00 |
|  | Cancer | 4,136 (7.6%) | 5,679 (8.6%) | 0.04 | 8.2% | 8.2% | 0.00 |
| Hospital ward on admission | General ward | 46,665 (85.3%) | 53,525 (81.3%) | 0.11 | 83.2% | 83.1% | 0.00 |
|  | Intensive care unit/step down unit | 8,067 (14.7%) | 12,273 (18.7%) |  | 16.8% | 16.9% |  |
| Key diagnosis on admission | Sepsis | 258 (0.5%) | 241 (0.4%) | −0.02 | 0.4% | 0.4% | 0.00 |
|  | Pneumonia | 3,253 (5.9%) | 4,085 (6.2%) | 0.01 | 6.0% | 6.0% | 0.00 |
| Other COVID-19 treatments at baseline | Anticoagulants | 38,550 (70.4%) | 50,268 (76.4%) | 0.14 | 73.7% | 73.6% | 0.00 |
|  | Convalescent plasma | 17 (0.0%) | 49 (0.1%) | 0.53 | 0.1% | 0.1% | 0.00 |
|  | Corticosteroids | 33,342 (60.9%) | 55,041 (83.7%) | 0.02 | 73.6% | 73.6% | 0.00 |
|  | Baricitinib | 2,359 (4.3%) | 2,511 (3.8%) | 0.06 | 4.1% | 4.2% | 0.00 |
|  | Tocilizumab | 1,034 (1.9%) | 1,804 (2.7%) | −0.03 | 2.4% | 2.4% | 0.00 |
|  | Oral antivirals | 1,090 (2.0%) | 165 (0.3%) | −0.17 | 0.9% | 0.9% | 0.00 |
| Baseline supplemental oxygen requirements | NSOc | 31,151 (56.9%) | 30,268 (46.0%) | 0.23 | 2.3% | 2.3% | 0.00 |
|  | LFO | 15,191 (27.8%) | 22,122 (33.6%) |  | 15.8% | 15.9% |  |
|  | HFO/NIV | 6,971 (12.7%) | 12,100 (18.4%) |  | 30.9% | 31.1% |  |
|  | IMV | 1,419 (2.6%) | 1,308 (2.0%) |  | 51.0% | 50.8% |  |
| Omicron period | Early (Dec 2021-Dec 2022) | 37,298 (68.1%) | 43,244 (65.7%) | 0.05 | 66.7% | 66.7% | 0.00 |
|  | Late (Jan 2023-Feb 2024) | 17,434 (31.9%) | 22,554 (34.3%) |  | 33.3% | 33.3% |  |
| Abbreviations: COPD, chronic obstructive pulmonary disease; COVID-19, coronavirus disease 2019; HFO/NIV, high flow oxygen/non-invasive ventilation; ICF, intermediate care facility; IMV, invasive mechanical ventilation; IPTW, inverse probability of treatment weighting; LFO, low flow oxygen; NSOc, no supplemental oxygen charges; PSM, propensity score matching; SMD, standardized mean difference; SNF, skilled nursing facility | | | | | | | |

# Supplementary Table 7. 14- and 28-day Mortality in Elderly Patients Hospitalized for COVID-19 Receiving Remdesivir vs no Remdesivir by Supplemental Oxygen Requirements: Inverse Probability of Treatment Weighting

|  | **aHR [95% CI]** | **P-value** |
| --- | --- | --- |
| **14-day mortality** |  |  |
| Overall Omicron | 0.75 [0.71 – 0.79] | <.0001 |
| NSOc | 0.73 [0.67 – 0.78] | <.0001 |
| SOc | 0.75 [0.70 – 0.79] | <.0001 |
| **28-day mortality** |  |  |
| Overall Omicron | 0.77 [0.74 – 0.81] | <.0001 |
| NSOc | 0.76 [0.71 – 0.82] | <.0001 |
| SOc | 0.76 [0.72 – 0.81] | <.0001 |

Cox Proportional Hazards model used to derive estimates adjusted for age, admission month, hospital ward on admission (ICU vs general ward, and time-varying treatment with other COVID-19 medications (baricitinib, tocilizumab, oral antivirals).

Abbreviations: aHR, adjusted hazard ratio; CI, confidence interval; COVID-19, coronavirus disease 2019; NSOc, no supplemental oxygen charges; SOc, supplemental oxygen charges

# Supplementary Table 8. 14- and 28-day Mortality in Elderly Patients Hospitalized for COVID-19 Receiving Remdesivir in the First Two Days vs no Remdesivir in the First Two Days by Supplemental Oxygen Requirements: Propensity Score Matching

|  | **N** | **aHR [95% CI]** | **P-value** |
| --- | --- | --- | --- |
| **14-day mortality** |  |  |  |
| Overall Omicron | 84,882 | 0.73 [0.70 – 0.77] | <.0001 |
| NSOc | 43,746 | 0.70 [0.65 – 0.76] | <.0001 |
| SOc | 41,136 | 0.74 [0.70 – 0.79] | <.0001 |
| **28-day mortality** |  |  |  |
| Overall Omicron | 84,882 | 0.75 [0.72 – 0.79] | <.0001 |
| NSOc | 43,746 | 0.73 [0.68 – 0.79] | <.0001 |
| SOc | 41,136 | 0.75 [0.71 – 0.80] | <.0001 |

Cox Proportional Hazards model used to derive estimates adjusted for age, admission month, hospital ward on admission (ICU vs general ward, and time-varying treatment with other COVID-19 medications (baricitinib, tocilizumab, oral antivirals).

Abbreviations: aHR, adjusted hazard ratio; CI, confidence interval; COVID-19, coronavirus disease 2019; NSOc, no supplemental oxygen charges; SOc, supplemental oxygen charges

# Supplementary Table 9. Demographics of Patients Hospitalized for COVID-19 Pneumonia December 2021-February 2024 Before and After Propensity Score Matching

| **Characteristic** | | **Before PSM** | | | **After PSM** | | |
| --- | --- | --- | --- | --- | --- | --- | --- |
|  |  | **No Remdesivir**  **n =44,176** | **Remdesivir**  **n =68,507** | **SMD** | **No Remdesivir**  **N=36,385** | **Remdesivir**  **N=36,385** | **SMD** |
| Age group, years | 18–49 | 3,874 (8.8%) | 7,136 (10.4%) | 0.05 | 3,006 (8.3%) | 3,006 (8.3%) | 0.00 |
|  | 50–64 | 9,536 (21.6%) | 15,682 (22.9%) |  | 7,888 (21.7%) | 7,888 (21.7%) |  |
|  | ≥65 | 30,766 (69.6%) | 45,689 (66.7%) |  | 25,491 (70.1%) | 25,491 (70.1%) |  |
| Gender | Female | 21,763 (49.3%) | 33,971 (49.6%) | −0.01 | 17,948 (49.3%) | 17,915 (49.2%) | 0.00 |
| Race | White | 33,733 (76.4%) | 52,877 (77.2%) | 0.11 | 28,228 (77.6%) | 28,218 (77.6%) | 0.00 |
|  | Black | 6,516 (14.8%) | 8,500 (12.4%) |  | 4,862 (13.4%) | 4,868 (13.4%) |  |
|  | Asian | 743 (1.7%) | 1,534 (2.2%) |  | 646 (1.8%) | 632 (1.7%) |  |
|  | Other | 3,184 (7.2%) | 5,596 (8.2%) |  | 2,649 (7.3%) | 2,667 (7.3%) |  |
| Ethnicity | Hispanic | 3,538 (8.0%) | 7,626 (11.1%) | 0.11 | 2,968 (8.2%) | 2,901 (8.0%) | 0.00 |
|  | Non-Hispanic | 37,478 (84.8%) | 56,716 (82.8%) |  | 31,004 (85.2%) | 31,059 (85.4%) |  |
|  | Unknown | 3,160 (7.2%) | 4,165 (6.1%) |  | 2,413 (6.6%) | 2,425 (6.7%) |  |
| Primary payor | Commercial | 6,498 (14.7%) | 12,064 (17.6%) | 0.10 | 5,680 (15.6%) | 5,623 (15.5%) | 0.05 |
|  | Medicare | 31,424 (71.1%) | 46,413 (67.7%) |  | 25,703 (70.6%) | 25,745 (70.8%) |  |
|  | Medicaid | 3,707 (8.4%) | 6,300 (9.2%) |  | 2,947 (8.1%) | 2,974 (8.2%) |  |
|  | Other | 2,547 (5.8%) | 3,730 (5.4%) |  | 2,055 (5.6%) | 2,043 (5.6%) |  |
| Admission source | Transfer from SNF or ICF | 1,349 (3.1%) | 2,302 (3.4%) | 0.02 | 1,124 (3.1%) | 1,117 (3.1%) | 0.00 |
| Hospital size, number of beds | <100 | 3,687 (8.3%) | 5,475 (8.0%) | 0.09 | 3,136 (8.6%) | 3,039 (8.4%) | 0.05 |
|  | 100–199 | 7,289 (16.5%) | 12,421 (18.1%) |  | 6,143 (16.9%) | 6,240 (17.1%) |  |
|  | 200–299 | 9,583 (21.7%) | 14,058 (20.5%) |  | 7,993 (22.0%) | 7,836 (21.5%) |  |
|  | 300–399 | 8,193 (18.5%) | 11,383 (16.6%) |  | 6,664 (18.3%) | 6,538 (18.0%) |  |
|  | 400–499 | 5,238 (11.9%) | 7,440 (10.9%) |  | 4,106 (11.3%) | 4,389 (12.1%) |  |
|  | 500+ | 10,186 (23.1%) | 17,730 (25.9%) |  | 8,343 (22.9%) | 8,343 (22.9%) |  |
| Hospital location | Urban | 38,158 (86.4%) | 60,200 (87.9%) | 0.04 | 31,497 (86.6%) | 31,546 (86.7%) | 0.00 |
|  | Rural | 6,018 (13.6%) | 8,307 (12.1%) |  | 4,888 (13.4%) | 4,839 (13.3%) |  |
| Teaching hospital | | 17,545 (39.7%) | 28,414 (41.5%) | 0.04 | 14,262 (39.2%) | 14,259 (39.2%) | 0.00 |
| Region | Midwest | 11,732 (26.6%) | 16,628 (24.3%) | 0.21 | 9,759 (26.8%) | 9,599 (26.4%) | 0.03 |
|  | Northeast | 3,941 (8.9%) | 10,057 (14.7%) |  | 3,528 (9.7%) | 3,612 (9.9%) |  |
|  | South | 23,195 (52.5%) | 32,929 (48.1%) |  | 18,520 (50.9%) | 18,658 (51.3%) |  |
|  | West | 5,308 (12.0%) | 8,893 (13.0%) |  | 4,578 (12.6%) | 4,516 (12.4%) |  |
| Comorbid conditions | Obesity | 13,691 (31.0%) | 22,276 (32.5%) | 0.03 | 11,514 (31.6%) | 11,485 (31.6%) | 0.00 |
|  | COPD | 15,709 (35.6%) | 26,270 (38.3%) | 0.06 | 13,347 (36.7%) | 13,387 (36.8%) | 0.00 |
|  | Cardiovascular disease | 39,008 (88.3%) | 58,605 (85.5%) | −0.08 | 31,847 (87.5%) | 31,888 (87.6%) | 0.00 |
|  | Diabetes | 18,242 (41.3%) | 26,747 (39.0%) | −0.05 | 14,714 (40.4%) | 14,716 (40.4%) | 0.00 |
|  | Renal disease | 15,387 (34.8%) | 16,745 (24.4%) | −0.23 | 11,533 (31.7%) | 11,420 (31.4%) | −0.01 |
|  | Immunocompromising condition | 7,607 (17.2%) | 11,899 (17.4%) | 0.00 | 6,304 (17.3%) | 6,288 (17.3%) | 0.00 |
|  | Cancer | 3,195 (7.2%) | 5,028 (7.3%) | 0.00 | 2,665 (7.3%) | 2,635 (7.2%) | 0.00 |
| Hospital ward on admission | General ward | 35,827 (81.1%) | 54,851 (80.1%) | 0.03 | 29,682 (81.6%) | 29,799 (81.9%) | −0.01 |
|  | Intensive care unit/step down unit | 8,349 (18.9%) | 13,656 (19.9%) |  | 6,703 (18.4%) | 6,586 (18.1%) |  |
| Key Diagnosis on admission | Sepsis | 216 (0.5%) | 252 (0.4%) | −0.02 | 162 (0.4%) | 163 (0.4%) | 0.00 |
|  | Pneumonia | 3,739 (8.5%) | 4,979 (7.3%) | −0.04 | 2,921 (8.0%) | 2,956 (8.1%) | 0.00 |
| Other COVID-19 treatments at baseline | Anticoagulants | 32,625 (73.9%) | 54,818 (80.0%) | 0.15 | 27,886 (76.6%) | 27,895 (76.7%) | 0.00 |
|  | Convalescent plasma | 28 (0.1%) | 88 (0.1%) | 0.38 | 26 (0.1%) | 26 (0.1%) | 0.00 |
|  | Corticosteroids | 34,581 (78.3%) | 62,866 (91.8%) | 0.02 | 31,657 (87.0%) | 31,657 (87.0%) | 0.00 |
|  | Baricitinib | 3,750 (8.5%) | 4,764 (7.0%) | 0.05 | 3,128 (8.6%) | 3,119 (8.6%) | 0.00 |
|  | Tocilizumab | 1,790 (4.1%) | 3,435 (5.0%) | −0.06 | 1,612 (4.4%) | 1,619 (4.4%) | 0.00 |
|  | Oral antivirals | 383 (0.9%) | 94 (0.1%) | −0.10 | 55 (0.2%) | 49 (0.1%) | 0.00 |
| Baseline supplemental oxygen requirements | NSOc | 18,833 (42.6%) | 25,573 (37.3%) | 0.16 | 14,771 (40.6%) | 14,771 (40.6%) | 0.00 |
|  | LFO | 14,919 (33.8%) | 25,080 (36.6%) |  | 13,047 (35.9%) | 13,047 (35.9%) |  |
|  | HFO/NIV | 8,338 (18.9%) | 15,726 (23.0%) |  | 7,324 (20.1%) | 7,324 (20.1%) |  |
|  | IMV | 2,086 (4.7%) | 2,128 (3.1%) |  | 1,243 (3.4%) | 1,243 (3.4%) |  |
| Omicron period | Early (Dec 2021-Dec 2022) | 34,953 (79.1%) | 53,056 (77.4%) | 0.04 | 29,230 (80.3%) | 29,230 (80.3%) | 0.00 |
|  | Late (Jan 2023-Feb 2024) | 9,223 (20.9%) | 15,451 (22.6%) |  | 7,155 (19.7%) | 7,155 (19.7%) |  |
| Abbreviations: COPD, chronic obstructive pulmonary disease; COVID-19, coronavirus disease 2019; HFO/NIV, high flow oxygen/non-invasive ventilation; ICF, intermediate care facility; IMV, invasive mechanical ventilation; IPTW, inverse probability of treatment weighting; LFO, low flow oxygen; NSOc, no supplemental oxygen charges; PSM, propensity score matching; SMD, standardized mean difference; SNF, skilled nursing facility | | | | | | | |

# Supplementary Table 10. Demographics of Patients Hospitalized for COVID-19 Pneumonia During December 2021-February 2024 Before and After Inverse Probability of Treatment Weighting

| **Characteristic** | | **Before IPTW** | | | **After IPTW** | | | |
| --- | --- | --- | --- | --- | --- | --- | --- | --- |
|  |  | **No Remdesivir**  **n = 44,176** | **Remdesivir**  **n = 68,507** | **SMD** | | **No Remdesivir** | **Remdesivir** | **SMD** |
| Age group, years | 18–49 | 3,874 (8.8%) | 7,136 (10.4%) | 0.05 | | 6.9% | 9.8% | 0.00 |
|  | 50–64 | 9,536 (21.6%) | 15,682 (22.9%) |  | | 20.5% | 22.4% |  |
|  | ≥65 | 30,766 (69.6%) | 45,689 (66.7%) |  | | 72.6% | 67.9% |  |
| Gender | Female | 21,763 (49.3%) | 33,971 (49.6%) | −0.01 | | 52.0% | 49.4% | 0.00 |
| Race | White | 33,733 (76.4%) | 52,877 (77.2%) | 0.11 | | 79.8% | 77.0% | 0.00 |
|  | Black | 6,516 (14.8%) | 8,500 (12.4%) |  | | 11.4% | 13.2% |  |
|  | Asian | 743 (1.7%) | 1,534 (2.2%) |  | | 2.4% | 2.0% |  |
|  | Other | 3,184 (7.2%) | 5,596 (8.2%) |  | | 6.5% | 7.8% |  |
| Ethnicity | Hispanic | 3,538 (8.0%) | 7,626 (11.1%) | 0.11 | | 7.7% | 9.9% | 0.00 |
|  | Non-Hispanic | 37,478 (84.8%) | 56,716 (82.8%) |  | | 85.9% | 83.6% |  |
|  | Unknown | 3,160 (7.2%) | 4,165 (6.1%) |  | | 6.4% | 6.5% |  |
| Primary payor | Commercial | 6,498 (14.7%) | 12,064 (17.6%) | 0.10 | | 4.3% | 16.5% | 0.00 |
|  | Medicare | 31,424 (71.1%) | 46,413 (67.7%) |  | | 91.3% | 69.1% |  |
|  | Medicaid | 3,707 (8.4%) | 6,300 (9.2%) |  | | 1.5% | 8.9% |  |
|  | Other | 2,547 (5.8%) | 3,730 (5.4%) |  | | 2.9% | 5.6% |  |
| Admission source | Transfer from SNF or ICF | 1,349 (3.1%) | 2,302 (3.4%) | 0.02 | | 4.7% | 3.2% | 0.00 |
| Hospital size, number of beds | <100 | 3,687 (8.3%) | 5,475 (8.0%) | 0.09 | | 8.2% | 8.2% | 0.00 |
|  | 100–199 | 7,289 (16.5%) | 12,421 (18.1%) |  | | 16.9% | 17.6% |  |
|  | 200–299 | 9,583 (21.7%) | 14,058 (20.5%) |  | | 21.0% | 21.1% |  |
|  | 300–399 | 8,193 (18.5%) | 11,383 (16.6%) |  | | 17.2% | 17.2% |  |
|  | 400–499 | 5,238 (11.9%) | 7,440 (10.9%) |  | | 11.4% | 11.3% |  |
|  | 500+ | 10,186 (23.1%) | 17,730 (25.9%) |  | | 25.3% | 24.6% |  |
| Hospital location | Urban | 38,158 (86.4%) | 60,200 (87.9%) | 0.04 | | 87.6% | 87.3% | 0.01 |
|  | Rural | 6,018 (13.6%) | 8,307 (12.1%) |  | | 12.4% | 12.7% |  |
| Teaching hospital | | 17,545 (39.7%) | 28,414 (41.5%) | 0.04 | | 41.50% | 40.7% | 0.00 |
| Region | Midwest | 11,732 (26.6%) | 16,628 (24.3%) | 0.21 | | 24.6% | 25.3% | 0.03 |
|  | Northeast | 3,941 (8.9%) | 10,057 (14.7%) |  | | 14.8% | 12.4% |  |
|  | South | 23,195 (52.5%) | 32,929 (48.1%) |  | | 48.8% | 49.6% |  |
|  | West | 5,308 (12.0%) | 8,893 (13.0%) |  | | 11.8% | 12.7% |  |
| Comorbid conditions | Obesity | 13,691 (31.0%) | 22,276 (32.5%) | 0.03 | | 22.5% | 32.0% | 0.00 |
|  | COPD | 15,709 (35.6%) | 26,270 (38.3%) | 0.06 | | 37.4% | 37.4% | 0.00 |
|  | Cardiovascular disease | 39,008 (88.3%) | 58,605 (85.5%) | −0.08 | | 93.4% | 86.6% | 0.00 |
|  | Diabetes | 18,242 (41.3%) | 26,747 (39.0%) | −0.05 | | 39.8% | 39.9% | 0.00 |
|  | Renal disease | 15,387 (34.8%) | 16,745 (24.4%) | −0.23 | | 33.7% | 28.6% | 0.00 |
|  | Immunocompromising condition | 7,607 (17.2%) | 11,899 (17.4%) | 0.00 | | 17.7% | 17.4% | 0.00 |
|  | Cancer | 3,195 (7.2%) | 5,028 (7.3%) | 0.00 | | 8.2% | 7.3% | 0.00 |
| Hospital ward on admission | General ward | 35,827 (81.1%) | 54,851 (80.1%) | 0.03 | | 83.2% | 80.5% | 0.00 |
|  | Intensive care unit/step down unit | 8,349 (18.9%) | 13,656 (19.9%) |  | | 16.8% | 19.5% |  |
| Key diagnosis on admission | Sepsis | 216 (0.5%) | 252 (0.4%) | −0.02 | | 0.4% | 0.4% | 0.00 |
|  | Pneumonia | 3,739 (8.5%) | 4,979 (7.3%) | −0.04 | | 6% | 7.60% | 0.00 |
| Other COVID-19 treatments at baseline | Anticoagulants | 32,625 (73.9%) | 54,818 (80.0%) | 0.15 | | 73.7% | 77.6% | 0.00 |
|  | Convalescent plasma | 28 (0.1%) | 88 (0.1%) | 0.38 | | 0.1% | 0.1% | 0.00 |
|  | Corticosteroids | 34,581 (78.3%) | 62,866 (91.8%) | 0.02 | | 73.6% | 86.5% | 0.00 |
|  | Baricitinib | 3,750 (8.5%) | 4,764 (7.0%) | 0.05 | | 4.1% | 7.6% | 0.00 |
|  | Tocilizumab | 1,790 (4.1%) | 3,435 (5.0%) | −0.06 | | 2.4% | 4.7% | 0.00 |
|  | Oral antivirals | 383 (0.9%) | 94 (0.1%) | −0.10 | | 0.9% | 0.4% | 0.01 |
| Baseline supplemental oxygen requirements | NSOc | 18,833 (42.6%) | 25,573 (37.3%) | 0.16 | | 2.3% | 3.7% | 0.02 |
|  | LFO | 14,919 (33.8%) | 25,080 (36.6%) |  | | 15.8% | 21.4% |  |
|  | HFO/NIV | 8,338 (18.9%) | 15,726 (23.0%) |  | | 30.9% | 35.5% |  |
|  | IMV | 2,086 (4.7%) | 2,128 (3.1%) |  | | 51.0% | 39.4% |  |
| Omicron period | Early (Dec 2021-Dec 2022) | 34,953 (79.1%) | 53,056 (77.4%) | 0.04 | | 66.7% | 78.0% | 0.00 |
|  | Late (Jan 2023-Feb 2024) | 9,223 (20.9%) | 15,451 (22.6%) |  | | 33.3% | 22.0% |  |
| Abbreviations: COPD, chronic obstructive pulmonary disease; COVID-19, coronavirus disease 2019; HFO/NIV, high flow oxygen/non-invasive ventilation; ICF, intermediate care facility; IMV, invasive mechanical ventilation; IPTW, inverse probability of treatment weighting; LFO, low flow oxygen; NSOc, no supplemental oxygen charges; PSM, propensity score matching; SMD, standardized mean difference; SNF, skilled nursing facility | | | | | | | | |

# Supplementary Table 11. 14- and 28-day Mortality in Patients Hospitalized for COVID-19 Pneumonia Receiving Remdesivir vs no Remdesivir by Supplemental Oxygen Requirements: Inverse Probability of Treatment Weighting

|  | **aHR [95% CI]** | **P-value** |
| --- | --- | --- |
| **14-day mortality** |  |  |
| Overall Omicron | 0.76 [0.72 – 0.80] | <.0001 |
| NSOc | 0.75 [0.69 – 0.81] | <.0001 |
| SOc | 0.75 [0.71 – 0.80] | <.0001 |
| **28-day mortality** |  |  |
| Overall Omicron | 0.78 [0.74 – 0.82] | <.0001 |
| NSOc | 0.78 [0.73 – 0.84] | <.0001 |
| SOc | 0.77 [0.73 – 0.82] | <.0001 |

Cox Proportional Hazards model used to derive estimates adjusted for age, admission month, hospital ward on admission (ICU vs general ward, and time-varying treatment with other COVID-19 medications (baricitinib, tocilizumab, oral antivirals).

Abbreviations: aHR, adjusted hazard ratio; CI, confidence interval; COVID-19, coronavirus disease 2019; NSOc, no supplemental oxygen charges; SOc, supplemental oxygen charges

# Supplementary Table 12. 14- and 28-day Mortality in Patients Hospitalized for COVID-19 Pneumonia Receiving Remdesivir in the First Two Days vs no Remdesivir in the First Two Days by Supplemental Oxygen Requirements: Propensity Score Matching

|  | **N** | **aHR [95% CI]** | **P-value** |
| --- | --- | --- | --- |
| **14-day mortality** |  |  |  |
| Overall Omicron | 79,428 | 0.76 [0.72 – 0.80] | <.0001 |
| NSOc | 32,264 | 0.74 [0.68 – 0.81] | <.0001 |
| SOc | 47,164 | 0.77 [0.72 – 0.81] | <.0001 |
| **28-day mortality** |  |  |  |
| Overall Omicron | 79,428 | 0.77 [0.73 – 0.80] | <.0001 |
| NSOc | 32,264 | 0.77 [0.71 – 0.83] | <.0001 |
| SOc | 47,164 | 0.76 [0.72 – 0.80] | <.0001 |

Cox Proportional Hazards model used to derive estimates adjusted for age, admission month, hospital ward on admission (ICU vs general ward, and time-varying treatment with other COVID-19 medications (baricitinib, tocilizumab, oral antivirals).

Abbreviations: aHR, adjusted hazard ratio; CI, confidence interval; COVID-19, coronavirus disease 2019; NSOc, no supplemental oxygen charges; SOc, supplemental oxygen charges.

# Supplementary Figure 1. Balance of Standardized Mean Difference Before and After Propensity Score Matching Among Adults Hospitalized for COVID-19


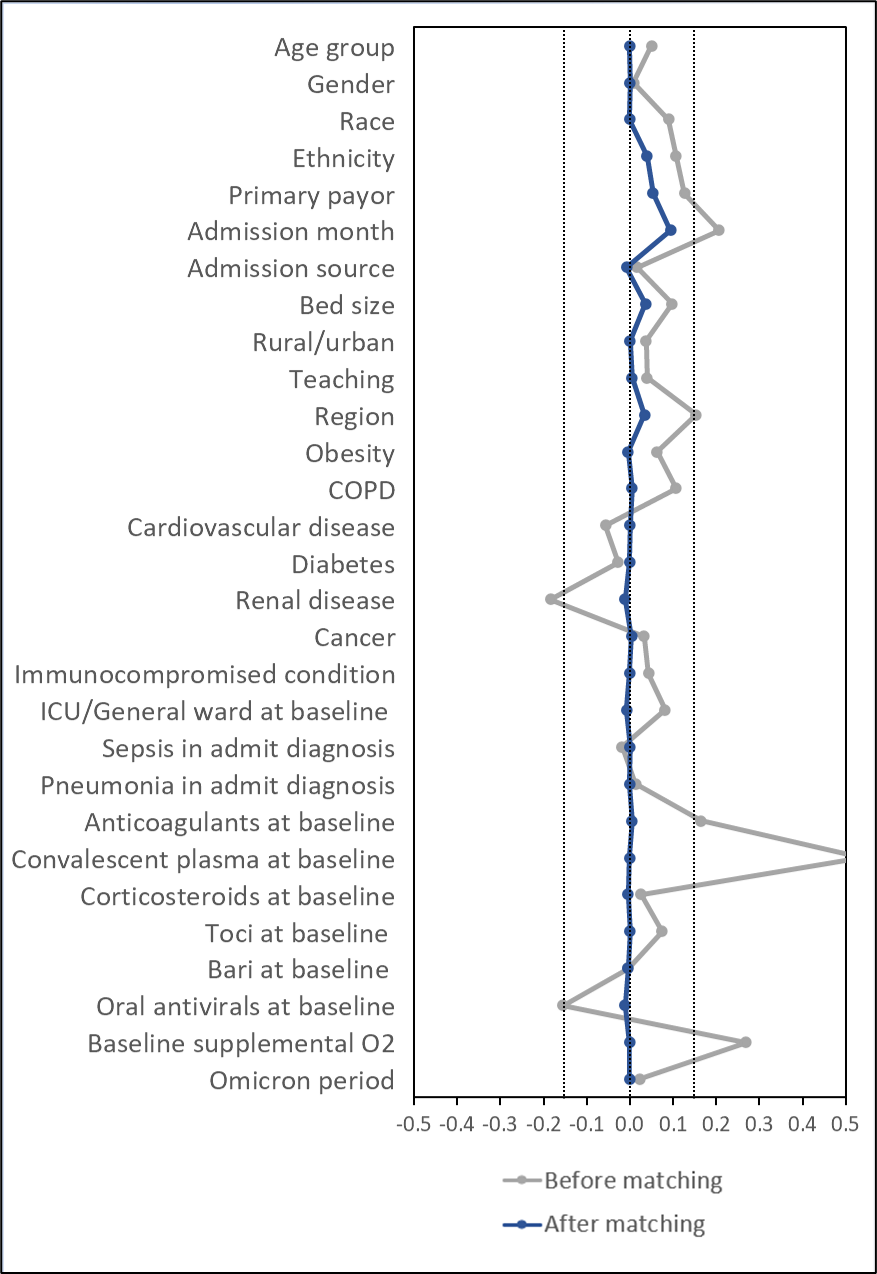


Abbreviations: COPD, Chronic Obstructive Pulmonary Disease; ICU, intensive care unit; O2, oxygen.

# Supplementary Figure 2. Balance of Standardized Mean Difference Before and After Propensity Score Matching Among Elderly Patients Hospitalized for COVID-19


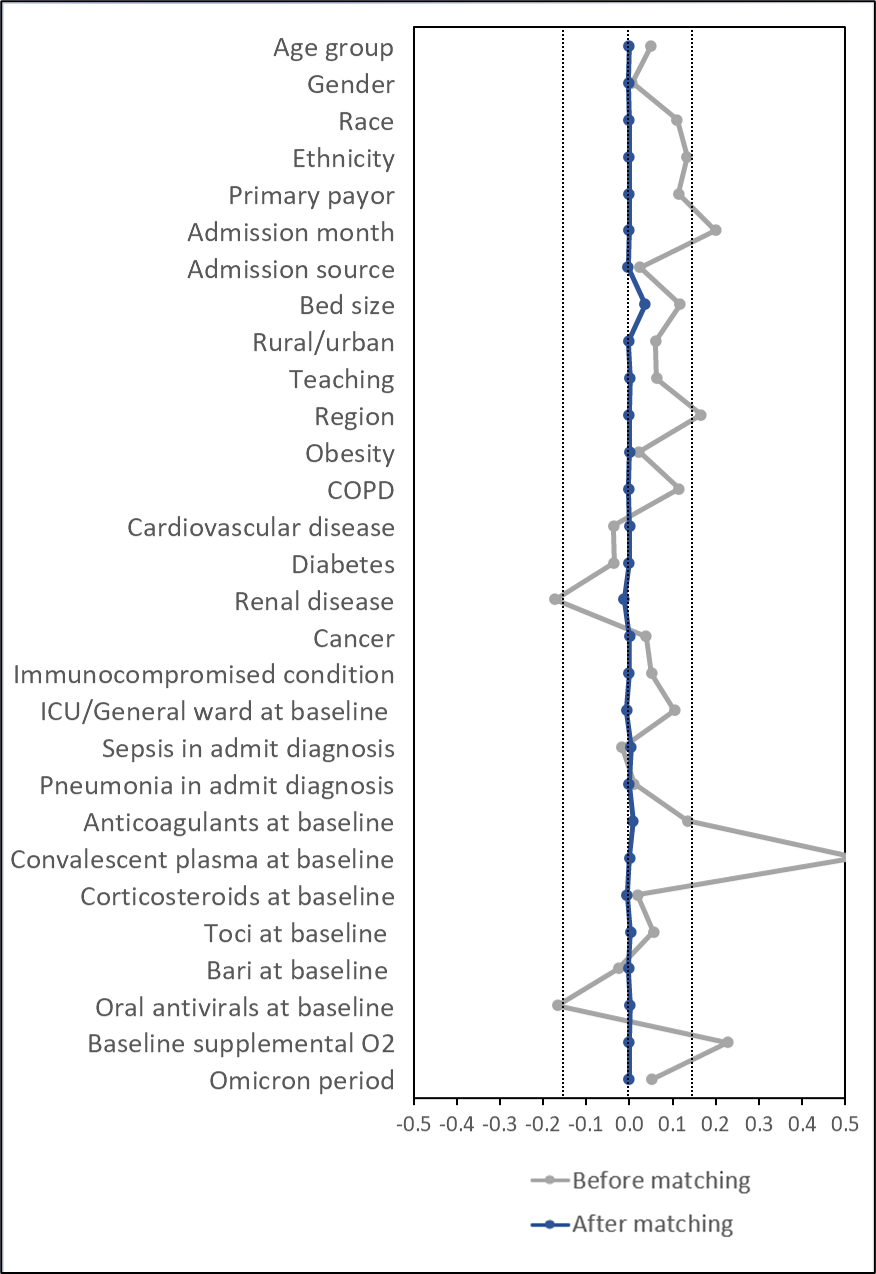


Abbreviations: COPD, Chronic Obstructive Pulmonary Disease; ICU, intensive care unit; O2, oxygen.

# Supplementary Figure 3. Balance of Standardized Mean Difference Before and After Propensity Score Matching Among Patients Hospitalized for COVID-19 Pneumonia


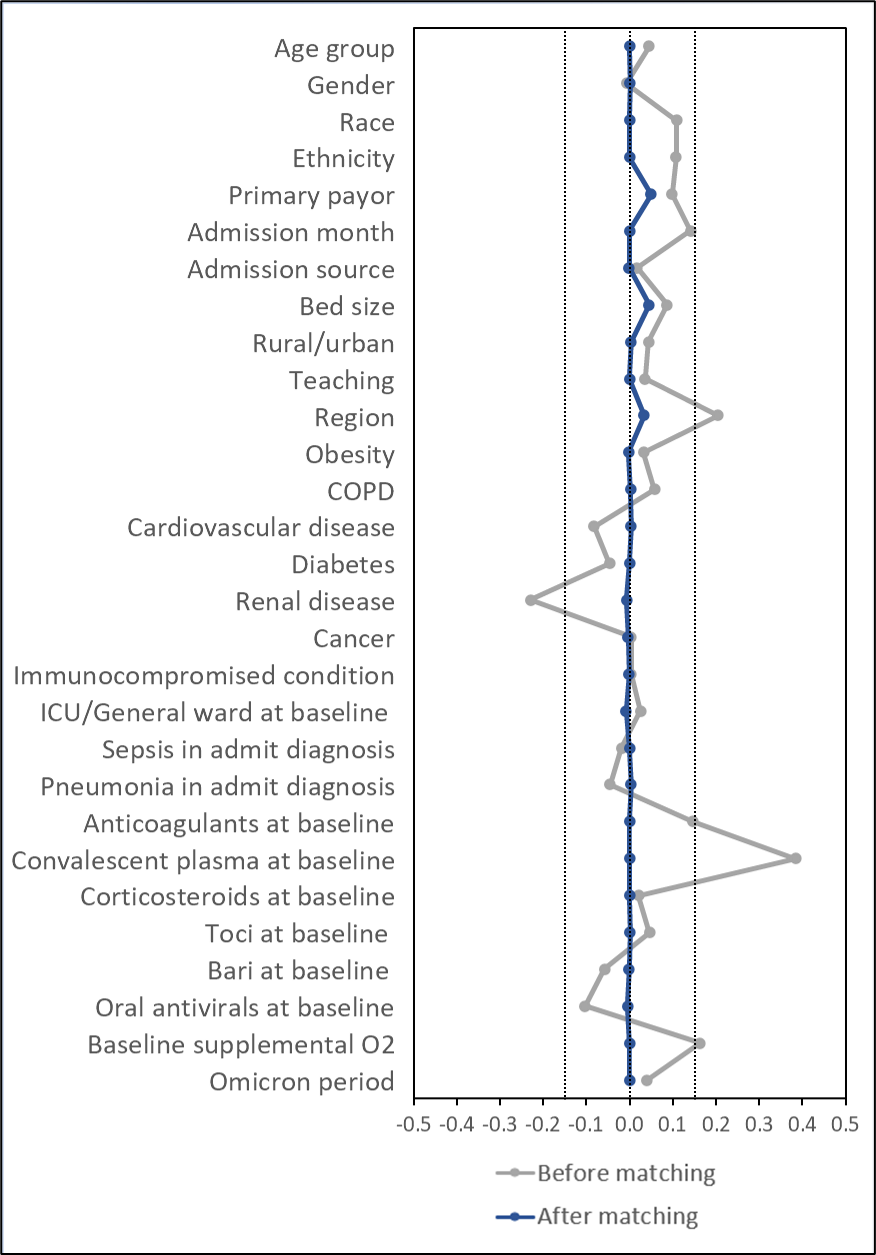


Abbreviations: COPD, Chronic Obstructive Pulmonary Disease; ICU, intensive care unit; O2, oxygen.
